# CsPbBr_3_ Nanocrystal Induced Bilateral Interface Modification for Efficient Planar Perovskite Solar Cells

**DOI:** 10.1002/advs.202102648

**Published:** 2021-09-13

**Authors:** Jianjun Zhang, Linxi Wang, Chenhui Jiang, Bei Cheng, Tao Chen, Jiaguo Yu

**Affiliations:** ^1^ State Key Laboratory of Advanced Technology for Materials Synthesis and Processing Wuhan University of Technology Wuhan 430070 P. R. China; ^2^ Laboratory of Solar Fuel Faculty of Materials Science and Chemistry China University of Geosciences Wuhan 430074 P. R. China; ^3^ Hefei National Laboratory for Physical Sciences at Microscale CAS Key Laboratory of Materials for Energy Conversion Department of Materials Science and Engineering School of Chemistry and Materials Science University of Science and Technology of China Hefei 230026 P. R. China

**Keywords:** built‐in electric field, CsPbBr_3_ nanocrystals, defect passivation, gradational incorporation, interface modification

## Abstract

Organic‐inorganic halide perovskite solar cells (PSCs) have drawn tremendous attention owing to their remarkable photovoltaic performance and simple preparation process. However, conventional wet‐chemical synthesis methods inevitably create defects both in the bulk and at the interfaces of perovskites, leading to recombination of charge carriers and reduced stability. Herein, a bilateral interface modification to perovskites by doping room‐temperature synthesized CsPbBr_3_ nanocrystals (CN) is reported. The ultrafast transient absorption measurement reveals that CN effectively suppresses the defect at the SnO_2_/perovskite interface and boosts the interfacial electron transport. Meanwhile, the in situ Kelvin probe force microscopy and contact potential difference characterizations verify that the CN within the upper part of the perovskites enhances the built‐in electric field, facilitating oriented migration of the carriers within the perovskite. Combining the superiorities of CN modifiers on both sides, the bilaterally modified CH_3_NH_3_PbI_3_‐based planar PSCs exhibit optimal power conversion efficiency exceeding 20% and improved device stability.

## Graphical abstract

Room‐temperature synthesized CsPbBr_3_ nanocrystal (CN) is exploited as the bilateral interface modifier in perovskite layer for efficient planar perovskite solar cells (PSCs). Owing to the CN induced bilateral interfacial passivation and boosted built‐in electric field, the charge separation and transfer are significantly ameliorated, which contribute to the superior power conversion efficiency exceeding 20% in CH_3_NH_3_PbI_3_‐based planar PSCs.

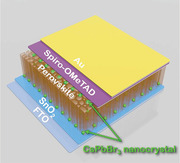

## 1 Introduction

Organic‐inorganic halide perovskite materials have shown immense potential in the emerging photovoltaic technology, owing to their prominent characteristics such as adjustable bandgap, high absorption coefficient, superb carrier mobility, and small exciton binding energy.^[^ ^1^, ^2^, ^3^ ^]^ Due to great research efforts devoted during the past decade, the power conversion efficiency (PCE) of perovskite solar cells (PSCs) have achieved impressive increase from 9.7% to 25.5%.^[^ ^4^, ^5^ ^]^ Nonetheless, there still exist great challenges to achieve desired PCEs, such as the formation of detrimental defects inside perovskite films and at interfaces during the fabrication of PSCs.^[^ ^6^, ^7^ ^]^ These defects serve as recombination sites for photogenerated electrons and holes, causing the loss of the effective carriers.^[^ ^8^ ^]^ In addition, the defects are highly sensitive to moisture, heat, oxygen, and ultraviolet light, leading to decomposition of the perovskite.^[^ ^9^, ^10^ ^]^ Therefore, defect control is crucial for fabrication of PSCs with high efficiency and long‐term stability. Multitudinous strategies have managed to reduce defect levels in perovskite films such as addictive passivation, heterojunction engineering, and interface modification.^[^ ^11^, ^12^, ^13^, ^14^ ^]^ These methods mainly focus on modification of perovskites using salts, polymers, and molecules. However, the modifiers have completely different components and structures from perovskites and hence are immiscible. In this regard, modifiers with better physicochemical compatibility with perovskite will be more desirable.

Recently, lead halide perovskite nanocrystals and quantum dots (QDs) have been widely utilized in the field of light‐emitting diodes, attributed to their high photoluminescence (PL) quantum yields (nearly 100%), outstanding processability, and simple preparation.^[^ ^15^, ^16^, ^17^ ^]^ The perovskite nanocrystals still retain the superb optoelectronic properties of their bulk counterpart and possess similar characteristics in stoichiometry and crystal structure.^[^ ^18^ ^]^ Hence, the perovskite nanocrystals can be a brilliant candidate for the modification in perovskite films and at interfaces. Previous studies have already verified the significance of perovskite nanocrystals and QDs in passivation. Concerning bottom modification of perovskite films, CsPbBr_3_ nanocrystals (CN) based interlayer could serve as the seed‐mediated layer for the subsequent crystallization of perovskite, leading to fewer defects in the perovskite and retarded charge recombination.^[^ ^19^ ^]^ As for the top modification, Cs_0.05_(MA_0.17_FA_0.83_)_0.95_PbBr_3_ QDs were introduced into the perovskite film through solid‐state interdiffusion process, which passivated the ionic defects at the surface and grain boundaries.^[^ ^20^ ^]^ CsPbBrCl_2_ QDs were exploited as the molecular surface modifiers, realizing decreased tail states and reduced trap‐state density.^[^ ^21^ ^]^ CN or nanowires based surface modifier successfully constructed a graded heterojunction at the upper part of perovskite film to ameliorate interfacial band alignment.^[^ ^22^, ^23^ ^]^ CsPbBrI_2_ QDs were utilized to construct a graded dimensional perovskite interface in all‐inorganic PSCs, contributing to enhanced hole extraction and conduction efficiency.^[^ ^24^ ^]^ However, it has been neglected that bottom and top modification of perovskite film by perovskite nanocrystals can be achieved simultaneously. Besides, compared to the conventional hot‐injection procedure, the preparation of perovskite nanocrystals can be simplified and conducted under much milder conditions.

In this work, CN is successfully synthesized via a simple one‐step injection method at room temperature and applied as the bilateral interface modifier for CH_3_NH_3_PbI_3_ (MAPbI_3_) perovskite film. To fulfill the CN bottom modification, CN is spin‐coated on the SnO_2_ electron transport layer (ETL). CN can serve as the seed crystal to facilitate the seed‐mediated growth of perovskite, which effectively passivates the SnO_2_ ETL/perovskite interface and reduces ionic defects in the perovskite film. Meanwhile, the CN can also decrease the energy barrier between the SnO_2_ ETL and the perovskite layer, enabling more efficient electron extraction. As for the CN top modification, toluene (TL) containing dispersed CN is utilized as the antisolvent during the spin‐coating process of the perovskite film to realize gradational incorporation of CN at the upper part of perovskite film. Apart from passivating the surface and strengthening the moisture resistance, CN top modification can also shift down the Fermi level at the perovskite surface and thus enlarge the difference between Fermi levels in the surface and bottom layers. The increased gap between Fermi levels reinforces the built‐in electric field, leading to boosted separation and oriented transport of carriers inside the perovskite film. Ultimately, by integrating the advantages in CN bottom and top modification, MAPbI_3_‐based planar PSCs with CN bilateral interface modification exhibit an average PCE of 20.1% and boosted device stability. Our findings have systematically revealed the functions of CN modification in the bilateral surface of perovskite film, which also provides guidance for the utilization of perovskite nanocrystals in perovskite‐based devices.

## 2 Results and Discussion

The CN was prepared through a simple and fast one‐step injection method in air at room temperature.^[^ ^25^ ^]^ The detailed synthesis process can be found in the Supporting Information. According to the transmittance electron microscopy (TEM) images (Figure S1a,b, Supporting Information), the as‐prepared CNs possess an average size of ≈10 nm and the lattice spacing in the high‐resolution TEM image corresponds to the (200) plane. The X‐ray diffraction (XRD) results in Figure S1c, Supporting Information further verify that CN is well‐crystallized into orthorhombic structure (PDF#18‐0364).^[^ ^26^ ^]^ As exhibited in Figure S1d, Supporting Information, the UV−vis absorption and PL emission spectra illustrate the absorption edge of CN at ≈524 nm, equivalent to a band gap of ≈2.37 eV.^[^ ^27^ ^]^ The CN suspension (in TL) emits bright green light under 365 nm light excitation, which matches the band gap of CN.^[^ ^28^ ^]^

**Figure 1:**
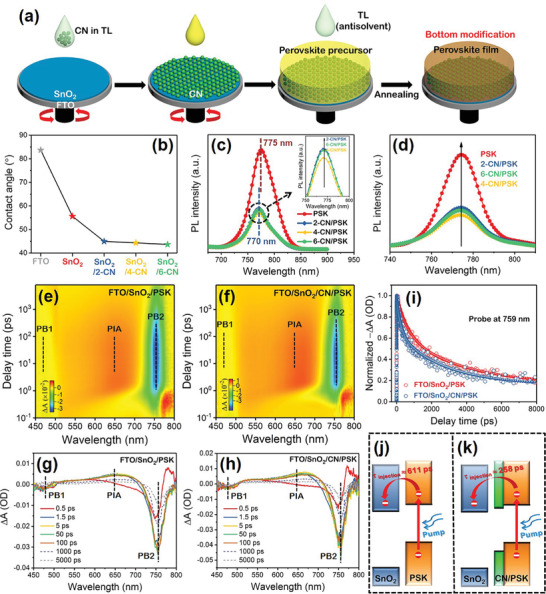
a) Schematic for CN induced bottom modification of perovskite film. b) The contact angles of water droplets on FTO and SnO_2_ without and with different times of CN modification. Steady‐state PL spectra of perovskite films deposited on CN modified SnO_2_ ETLs illuminated from c) the FTO‐glass side and d) perovskite film side. e,f) The pseudocolor plots, g,h) transient TA spectra of FTO/SnO_2_/PSK and FTO/SnO_2_/CN/PSK excited at 400 nm, and i) corresponding normalized decay kinetic curves at 759 nm. Schematics for electron generation and extraction at the interface j) between SnO_2_ and PSK or k) between SnO_2_ and CN/PSK.

Considering the superior dispersibility of CN in TL, we prepare CN/TL suspension for modification of perovskite films. **Figure** **1**a depicts the steps of the CN bottom modification of pristine perovskite film (PSK). The CN is introduced onto the SnO_2_ ETL via spin‐coating a CN suspension for two, four, and six times. All the solvents and ligands in the suspension evaporate quickly during the spin‐coating. The obtained SnO_2_ ETLs after different cycles of spin‐coating are marked as SnO_2_/2‐CN, SnO_2_/4‐CN, and SnO_2_/6‐CN, while the MAPbI_3_ perovskite films subsequently deposited on these ETLs are respectively labeled as 2‐CN/PSK, 4‐CN/PSK, and 6‐CN/PSK. The surface morphology of the SnO_2_ ETL hardly changes with increasing times of CN bottom modification, but the root‐mean‐square roughness (R_q_) of SnO_2_ ETLs gradually enlarges upon the introduction of CNs (Figures S2–S4, Supporting Information). As shown in Figures S3 and S5, Supporting Information, CN begins to aggregate after six cycles of CN bottom modification, leading to the nonuniform SnO_2_ surface. The agglomerating CNs will influence the normal crystallization process of perovskite, which is disadvantageous to the uniformity of perovskite layer. In addition, EDS mappings of Cs, Pb, and Br in the SnO_2_/4‐CN film reveal that the CNs are still uniformly distributed on the SnO_2_ surface after four times of CN bottom modification (Figures S6 and S7, Supporting Information). The XRD characterizations in Figure S8a, Supporting Information further confirm the presence of CN on the SnO_2_ film. During the wavelength region of 300–500 nm, the transmittance of the SnO_2_ films decreases with increasing contents of CN (Figure S8b, Supporting Information). This suggests that CN modified SnO_2_ films can absorb certain amount of ultraviolet (UV) light, which is potentially useful in alleviating decomposition of the perovskite film induced by UV radiation.^[^ ^29^ ^]^

After CN bottom modification, the contact angles for SnO_2_ films decrease from ≈55° to ≈45° (Figure S9, Supporting Information and Figure 1b), which is beneficial for spreading the perovskite precursor solution onto the SnO_2_ ETL.^[^ ^30^ ^]^ During the spreading of perovskite precursor, the CN on the surface of SnO_2_ film are partially dissolved by N,N‐dimethylformamide and dimethyl sulfoxide. Dissolved CsPbBr_3_ will recrystallize and incorporate into the MAPbI_3_ lattice at the bottom of perovskite film. The perovskite film grown on the CN modified SnO_2_ ETL has a nearly identical morphology to that on the pristine SnO_2_ ETL (Figure S10, Supporting Information), but its surface roughness slightly decreases (Figures S11–S13, Supporting Information). The XRD patterns in Figure S14a, Supporting Information further prove high crystallinity of the deposited perovskite films on the CN modified SnO_2_ ETLs. The slightly enhanced absorbance in the visible light region (Figure S14b, Supporting Information) also confirms improved crystallinity after CN bottom modification.^[^ ^31^ ^]^ Previous article has reported that the uniformly distributed nanocrystals are capable of adjusting the nucleation rate and new phase formation of the perovskite film.^[^ ^32^ ^]^ Hence, all the aforementioned improvements can be attributed to the reason that CN serves as the seed crystal to impel seed‐mediated growth of the perovskite, which decreases the interfacial trap sites and reduces defects inside the perovskite film.^[^ ^33^ ^]^

The electron extraction capability between the SnO_2_ ETL and the perovskite film is characterized by steady‐state PL and time‐resolved transient PL (TRPL) on the FTO/SnO_2_/CN/PSK sample. When all the samples are illuminated from the FTO‐glass side, a blue shift of the PL peak is observed for the samples with CN bottom modification (Figure 1c), which does not happen when the samples are illuminated from the perovskite film side (Figure 1d). The blue shift reveals that the CN has directly become the structural component at the bottom of perovskite film. In comparison to pristine PSK, the 4‐CN/PSK possesses the lowest PL intensity and shorter average lifetime (Figure S14c, Supporting Information), indicating more efficient electron transfer after moderate CN bottom modification.^[^ ^34^, ^35^, ^36^ ^]^ This is due to the capability of CN to reduce the ionic defects and suppress the interfacial charge recombination between SnO_2_ ETL and perovskite film. The energy level alignment is another essential factor for efficient electron extraction.^[^ ^37^, ^38^ ^]^ Based on the information of the ultroviolet photoelectron spectra (UPS) in Figure S15a–d, Supporting Information, the position distributions of the conduction bands (CB), valence bands, and Fermi levels (*E*_f_) for PSK and CN are calculated (Figure S15e, Supporting Information).^[^ ^39^ ^]^ As shown in Figure S15f, Supporting Information, the CB of CN is located between the CB of SnO_2_ and MAPbI_3_ perovskite, implying CN bottom modification is able to decrease the energy barrier between the SnO_2_/perovskite interface and reduce the energy loss during the electron transport.^[^ ^40^, ^41^ ^]^

To further understand the kinetics of charge transfer at the SnO_2_/perovskite interface, ultrafast transient absorption (TA) characterizations are conducted on the FTO/SnO_2_(CN)/PSK sample with a 400 nm pump laser pulse. Figure 1e–h exhibits the representative pseudocolor 2D and transient TA spectra at different delay times. Two photobleaching (PB, ΔA < 0) signals and one photoinduced absorption (PIA, ΔA > 0) signal can be distinguished. The broad positive ΔA peak (PIA) from 550 to 600 nm corresponds to the absorption of certain transient species.^[^ ^42^ ^]^ The negative ΔA peaks at ≈480 nm (PB1) originate from the integration of PB and stimulated emission signals.^[^ ^43^ ^]^ Importantly, the PB2 signals approach to the absorption edge of MAPbI_3_ and reflect the photo‐induced carrier population in the MAPbI_3_, which can be well utilized to analyze the process of exciton generation and transport. The PB2 peak emerges quickly in just 1 ps (Figure S16, Supporting Information), because the MAPbI_3_ possesses the Wannier–Mott type excitons with low binding energy and produces free charge carriers with high generation rate.^[^ ^44^ ^]^ Owing to the hot carrier cooling, the PB2 peak rapidly intensifies within 5 ps.^[^ ^45^ ^]^ The following relaxation of the excited carriers is basically divided into two representative processes: solid lines in Figure 1e,f are ascribed to the bimolecular recombination induced pump‐fluence dependent lifetime during a few hundred picoseconds, and the dotted lines in Figure 1e,f belong to the trap‐assisted monomolecular recombination during thousands of picoseconds.^[^ ^46^ ^]^ Furthermore, the normalized decay kinetic curves at 759 nm are finely fitted by a bi‐exponential function (Figure 1i) and their corresponding kinetic parameters are listed in Table S1, Supporting Information. The longer decay time (*τ*_2_) represents the excited‐state decay or carrier recombination in perovskite films, while the shorter decay time (*τ*_1_) reflects the electron injection from photoexcited MAPbI_3_ to the CB of SnO_2_.^[^ ^47^, ^48^ ^]^ Consequently, the electron injection time (*τ*_injection_) for the pristine PSK is calculated to be ≈611 ps (Figure 1j), which remarkably decreases to ≈258 ps with CN bottom modification (Figure 1k). The reduced *τ*_injection_ verifies that CN bottom modification can effectively passivate the SnO_2_/perovskite interfacial defects and expedite interfacial electron transport.

Finally, the planar PSCs with the structure of FTO/SnO_2_/MAPbI_3_/Spiro‐OMeTAD/Au are fabricated, and improved photovoltaic performance is observed after CN bottom modification (Figure S17a, Supporting Information). Benefiting from reduced defects and boosted electron extraction at the SnO_2_/perovskite interface, the 4‐CN/PSK based PSCs demonstrate an average PCE of 19.4%, higher than ≈18.2% for pristine PSCs (Figure S17b and Table S2, Supporting Information). The PCE of the 6‐CN/PSK based PSCs decreases owing to increased defects caused by agglomeration of the CN. Besides, electrochemical impedance spectroscopy (EIS) under dark condition (Figure S17c, Supporting Information) shows that the 4‐CN/PSK based PSC possesses the largest semicircle, which also reveals that the charge recombination is significantly retarded inside the device.^[^ ^49^ ^]^ In conclusion, CN bottom modification can successfully ameliorate the interfacial contact between the SnO_2_ ETL and the perovskite film.

**Figure 2:**
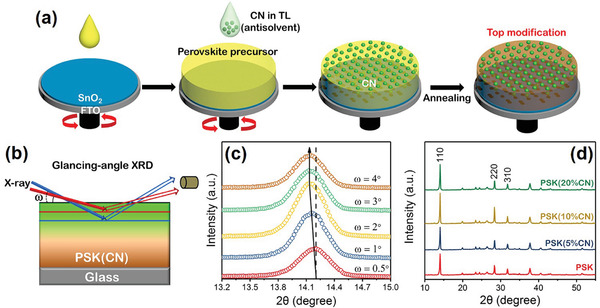
a) Schematic for CN induced top modification of perovskite film. b) Schematic of glancing‐angle XRD test. c) Glancing‐angle XRD patterns of perovskite film with CN top modification in different glancing angles. d) XRD patterns of perovskite films with different concentrations of CN top modification.

In addition to controlling defects at the bottom of perovskite film, CN also possesses great potential as a surface modifier for perovskite films. The schematic of CN top modification is presented in **Figure** **2**a. During the spin‐coating process of perovskite, TL with dispersed CN is utilized as the antisolvent. The antisolvent is diluted to 5%, 10%, and 20% of its original concentrations to deposit the perovskite film, which are respectively marked as PSK(5%CN), PSK(10%CN), and PSK(20%CN). The distribution of CN within the perovskites is characterized by the glancing‐angle XRD, whose schematic diagram is depicted in Figure 2b. X‐ray with different glancing‐angle (*ω*) is utilized to obtain the 2*θ* information for the (110) lattice plane at various depths. An increasing *ω* allows probing into deeper regions. With *ω* increasing from 0.5° to 2°, the position of the (110) peak gradually shifts to lower 2*θ* angle, suggesting a gradual expansion of lattice from the surface to the interior of the perovskite film (Figure 2c).^[^ ^23^ ^]^ This phenomenon also elucidates that the doping concentration of CN gradually decreases with increasing depth into the perovskite film. When *ω* increases from 2° to 4°, the 2*θ* angle for the (110) peak barely changes, illustrating that little CN exists at the bottom of the perovskite film. Therefore, a gradational incorporation of CN is constructed at the upper part of perovskite film. The surface roughness of the perovskite films slightly increases with CN top modification, while the typical compact and polycrystalline grain morphology remain (Figures S18–S20, Supporting Information). Meanwhile, the size of perovskite grains slightly decreases and the surface undulation mildly increases (Figure S21, Supporting Information). This can be interpreted as CN provides additional heterogeneous nucleation sites for the crystallization of MAPbI_3_ perovskite.^[^ ^50^, ^51^ ^]^ As exhibited in Figure 2d, the peak intensity of (110) and (220) lattice plane is significantly enhanced with moderate CN top modification, and PSK(10%CN) sample possesses the strongest peak intensity. This confirms that CN facilitates the formation of ordered perovskite crystalline structure that is preferentially oriented along the (110) lattice plane, which is advantageous to the charge transport within the perovskite film.^[^ ^52^ ^]^ Thanks to improved crystallinity of the perovskite film, the light absorbance is also enhanced (Figure S22a, Supporting Information). Compared to the PSK, the PSK(10%CN) exhibits higher steady‐state PL intensity (Figure S22b, Supporting Information) and longer average lifetime both in TRPL test (Figure S22c, Supporting Information) and TA measurement (Figure S23 and Table S3, Supporting Information), elucidating that non‐radiative recombination sites are effectively minimized. The cations and anions from the CN can occupy the ionic vacancies at the upper part of MAPbI_3_ film through solid‐state interdiffusion, so the ionic defects at the surface and grain boundaries are significantly repaired.^[^ ^20^ ^]^ Ultimately, the PSK(10%CN) based PSC shows an average PCE of 18.9% with increased open‐circuit voltage (*V*_oc_) and short‐circuit current (*J*_sc_) (Figure S24a,b and Table S4, Supporting Information), attributed to the ameliorative quality of the perovskite film and suppressed charge recombination (Figure S24c, Supporting Information). Consequently, the CN top modification is proved to be a brilliant ionic defect passivation strategy for perovskite films.

**Figure 3:**
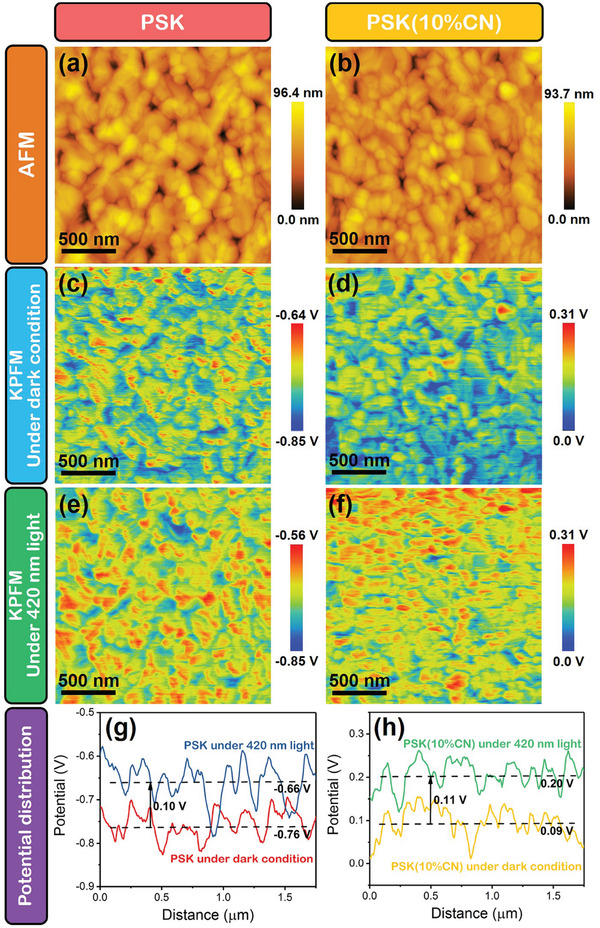
a,b) AFM images of PSK and PSK(10%CN). In situ KPFM images of PSK and PSK(10%CN) under c,d) dark condition and e,f) 420 nm light illumination. g,h) Surface potential distributions of PSK and PSK(10%CN) under dark condition and 420 nm light illumination.

Another effect of CN top modification is tuning the surface potential of perovskite films, which can be investigated by the Kelvin probe force microscopy (KPFM).^[^ ^53^ ^]^ **Figure** **3**a,b exhibits the surface morphologies of PSK and PSK(10%CN), and the corresponding in situ KPFM images under dark condition or 420 nm light illumination are displayed in Figure 3c–f. It is obvious that the PSK(10%CN) possesses much higher surface potential than PSK under both dark condition or 420 nm light illumination. Under both conditions, the surface potential of the PSK(10%CN) remains evenly distributed, suggesting that CNs have introduced uniform doping near the surface of PSK(10%CN). The specific values of surface potentials are obtained from linear distribution as shown in Figure 3g,h. Under dark condition, the CN top modification significantly upshifts the surface potential from −7.6 to 0.09 V, indicating a deeper Fermi level is constructed on the surface of PSK (10%CN). With regard to the regular n–i–p structured PSCs, deeper Fermi level on the surface of perovskite may reduce *V*_oc_ loss and facilitate the hole transport from the perovskite film to the hole transport layer (HTL).^[^ ^54^ ^]^ Under irradiation of the 420 nm light, an increased surface potential and a positive surface photovoltage (SPV) are observed in both the PSK and PSK(10%CN). This is because the as‐prepared PSK and PSK(10%CN) are both weak p‐type semiconductor, as confirmed by the UPS results in Figures S15 and S25, Supporting Information.^[^ ^55^ ^]^ Furthermore, the SPV of PSK(10%CN) film (≈0.11 V) is close to that of the PSK film (≈0.10 V), indicating that the deeper Fermi level of PSK(10%CN) surface is retained under illumination. Therefore, this superiority of PSK(10%CN) is also useful for improving the photovoltaic performance of complete PSC.

**Figure 4:**
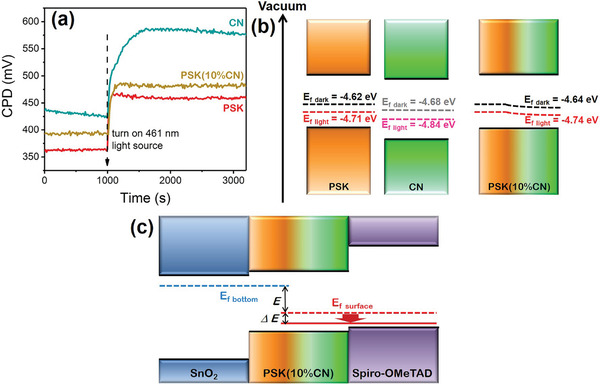
a) In situ CPD detection of PSK, CN, and PSK (10% CN) under dark condition and 461 nm light illumination. b) Schematic for the Fermi level of PSK, CN, and PSK(10%CN) under dark condition and 461 nm light illumination. c) Schematic for the changes of Fermi level at the interfaces of PSK/ETL and PSK/HTL.

To acquire more specific information, the average surface work function (*W*) of the perovskite films is measured by the Kelvin probe instrument. **Figure** **4**a exhibits the stable contact potential difference (CPD) values of the PSK, PSK(10%CN), and CN under dark condition and 461 nm light illumination. The related parameters including work function (*W*), Fermi level (*E*_f_), and SPV can be calculated through the following equations:^[^ ^56^ ^]^

(1) $$\mathrm{SPV}\;=\;\mathrm{CP}D_{\mathrm{illumination}}\;-\mathrm{CP}D_{\mathrm{dark}}$$

(2) $$W=W_{\mathrm{tip}}+e\cdot \mathrm{CP}D_{\mathrm{sample}}$$

(3) $$E_{f}=-W$$

where CPD_illumination_ and CPD_dark_ represent the CPD of samples under dark condition and 461 nm light illumination, *e* is the electron charge, and *W*_tip_ is the standard *W* of the gold probe (established to be 4.25 eV). Results of these surface characteristic parameters are listed in **Table** **1**. The PSK and PSK(10%CN) have similar SPV, which is in consistent with the aforementioned KPFM results. According to the position of Fermi levels in PSK, CN, and PSK(10%CN), a schematic of Fermi level distributions is presented in Figure 4b. The Fermi level of the PSK is higher than that of the CN under both dark conditions and 461 nm light illumination. As discussed above, CN top modification can achieve a gradational incorporation of CN at the upper part of the perovskite film and the doping concentration of CN gradually decays at deeper regions. Hence, a gradual downshift of Fermi level near the surface of PSK(10%CN) film is proposed. According to the CPD results, the Fermi level of PSK(10%CN) surface under dark condition and 461 nm light illumination is calculated to be −4.64 and −4.74 eV respectively, lower than the Fermi level of PSK surface. This is consistent with the aforementioned UPS results. As shown in Figure 4c, when the perovskite film is sandwiched between SnO_2_ ETL and Spiro‐OMeTAD HTL, a stabilized Fermi level is formed at the bottom (*E*_f bottom_) and surface (*E*_f surface_) of perovskite film after interfacial charge transfer.^[^ ^57^, ^58^ ^]^ The difference in Fermi levels (*E*) at the bottom and surface of the perovskite film is the source of the built‐in electric field, which is the driving force for the separation of electrons and holes inside perovskite film.^[^ ^59^ ^]^ Hence, the built‐in electric field can be enlarged by increasing the difference in Fermi levels.^[^ ^60^ ^]^ With CN top modification, an increased difference in Fermi levels (*ΔE*) is achieved under both dark and illuminated conditions, resulting in a stronger built‐in electric field that better orients charge transfers to further enhance the photovoltaic performance of PSCs. In conclusion, the ameliorative oriented charge transfer also contributes to the optimal PCE of PSK(10%CN) based PSCs.

**Table 1:** Surface characteristic parameters obtained from CPD

Samples	PSK		PSK(10%CN)		CN	
Conditions	Dark	Illumination	Dark	Illumination	Dark	Illumination
CPD [mV]	365	456	393	485	430	585
SPV [mV]	91		92		155	
*W* [eV]	4.62	4.71	4.64	4.74	4.68	4.84
*E*_f_ [eV]	−4.62	−4.71	−4.64	−4.74	−4.68	−4.84

**Figure 5:**
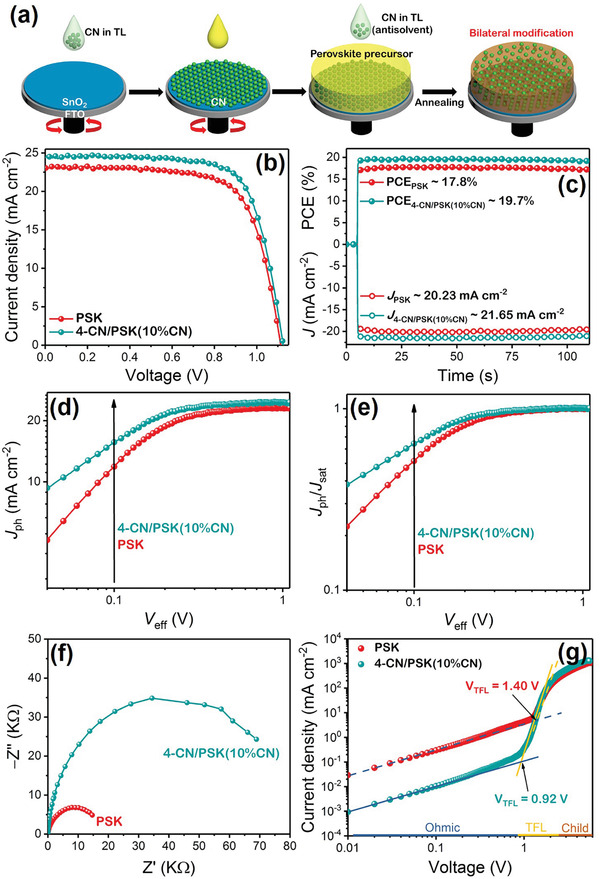
a) Schematic for CN bilateral interface modification of perovskite film. Characterizations of PSK and 4‐CN/PSK(10%CN) based planar PSCs: b) *J*–*V* curves under simulated AM 1.5 illumination; c) steady‐state photocurrent and PCE measured at the maximum power point; d) plots of photocurrent density (*J*_ph_) with regard to the effective bias (*V*_eff_); e) normalized photocurrent (*J*_ph_/*J*_sat_) with regard to *V*_eff_; f) EIS plots tested under dark condition. g) *J*–*V* curves of the electron‐only devices based on PSK and 4‐CN/PSK(10%CN) active layer.

In order to study the synergy of CN bottom and top modification, the planar PSCs with bilateral interface modification are fabricated (**Figure** **5**a). The optimal concentration of CN bottom modification and top modification is chosen for the bilateral interface modification, and the obtained bilaterally modified perovskite film is labeled as 4‐CN/PSK(10%CN). The photovoltaic performance of the planar PSCs composed of FTO/SnO_2_/4‐CN/PSK(10%CN)/Spiro‐OMeTAD/Au has also been investigated (Figure 5b) with corresponding photovoltaic parameters listed in **Table** **2**. The 4‐CN/PSK(10%CN) based planar PSCs demonstrate an average PCE of 20.1%, higher than that of the PSK, 4‐CN/PSK, and PSK(10%CN) based PSCs. All the related photovoltaic parameters including *V*_oc_, *J*_sc_, and *FF*, are improved through bilateral interface modification. Especially, the incident photon‐to‐current conversion efficiency results in Figure S26a, Supporting Information further confirm that 4‐CN/PSK(10%CN) based PSC displays a reinforced photoelectric response during the wavelength of 400–700 nm. This distinguished photovoltaic performance results from the combined effects of higher quality of perovskite film and more efficient charge extraction. To further verify the enhancement of PCE, stabilized photocurrent density and power output with a bias of maximum power point (MPP) voltage [0.88 V for PSK and 0.91 V for 4‐CN/PSK(10%CN)] are exhibited in Figure 5c. The 4‐CN/PSK(10%CN) based PSC demonstrates excellent *J*_sc_ of 21.65 mA cm^−2^ and PCE of 19.7% at steady state, whereas the pristine PSK based PSC only shows inferior *J*_sc_ of 20.23 mA cm^−2^ and PCE of 17.8%. The results of the stabilized PCE are close to the values extracted from the *J*–*V* curve. On the basis of the *J*–*V* curves under illumination (Figure 5b) and dark condition (Figure S26b, Supporting Information), the exciton dissociation probabilities in PSK and 4‐CN/PSK(10%CN) based PSCs can be analyzed by photocurrent density (*J*_ph_) and effective voltage (*V*_eff_).^[^ ^61^ ^]^ They are calculated through the following formulas:^[^ ^62^ ^]^

(4) $$J_{\mathrm{ph}}=J_{L}-J_{D}$$

(5) $$V_{\mathrm{eff}}=V_{0}-V$$

where *J*_L_ and *J*_D_ are the current density under illumination and dark condition; *V*_0_ represents the compensation voltage when *J*_ph_ = 0 and *V* signifies the applied voltage bias. As reflected in Figure 5d, *J*_ph_ increases sharply at the low *V*_eff_ and attains a saturated photocurrent (*J*_sat_) at high *V*_eff_. The 4‐CN/PSK(10%CN) based PSC reaches *J*_sat_ earlier than the pristine PSK based PSC, elucidating that more efficient charge separation is achieved through bilateral interface modification. In addition, the light‐harvesting ability of perovskite layer can be revealed via the maximum exciton‐generation rate (*G*_max_), as obtained by the following equation:^[^ ^63^ ^]^

(6) $$G_{\max}=\frac{J_{\mathrm{sat}}}{q\cdot L}$$

where *q* represents the charge quantity of one electron (1.6 × 10^−19^ C) and *L* is the thickness of perovskite film (≈400 nm). Intriguingly, the 4‐CN/PSK(10%CN) demonstrates superior *G*_max_ of 3.92 × 10^27^ m^−3^ s^−1^, whereas pristine PSK film exhibits inferior *G*_max_ of 3.51 × 10^27^ m^−3^ s^−1^. The boosted *G*_max_ also indicates that the light harvest and utilization of perovskite film are effectively improved through bilateral interface modification. Moreover, the normalized photocurrent density *J*_ph_/*J*_sat_ in Figure 5e can manifest the exciton dissociation probability (*P*).^[^ ^64^ ^]^ When *V*_eff_ is 0.1 V, the *P* values of PSK and 4‐CN/PSK(10%CN) based PSCs are 50% and 66%, respectively. The increased probability implies the exciton recombination is suppressed inside 4‐CN/PSK(10%CN) based PSC, consequently proving that CN bilateral interface modification contributes to more efficient charge separation and extraction. An enlarged semicircle is observed for the 4‐CN/PSK(10%CN) based PSC in the EIS test (Figure 5f), illustrating the recombination of carriers is effectively suppressed owing to the reformative interface and decreased defects induced by the CN bilateral interface modification. To measure the trap density of the perovskite films, space charge limited current (SCLC) test is conducted on a device composed of FTO/SnO_2_/perovskite/PCBM/Au. The trap‐filled limit voltage (*V*_TFL_) is acquired through the intersection voltage of ohmic and trap‐filled limit region, and the trap‐state density (*N*_traps_) can be determined by the following equation:^[^ ^65^, ^66^ ^]^

(7) $$N_{\mathrm{traps}}=\frac{2\varepsilon_{0}\varepsilon_{r}V_{\mathrm{TFL}}}{qL}$$

where *ɛ*_0_ signifies the vacuum permittivity, *ɛ*_r_ represents the relative dielectric constant of MAPbI_3_. The *N*_traps_ of PSK and 4‐CN/PSK(10%CN) are calculated to be 3.10 × 10^16^ cm^−3^ and 2.04 × 10^16^ cm^−3^, respectively. The reduced *N*_traps_ intuitively proves that the defects inside the perovskite film and at the interfaces are significantly passivated through CN bilateral interface modification.

**Table 2:** Detailed photovoltaic parameters for PSCs based on diverse CN modification (25 devices are fabricated for each sample)

Samples	*V*_oc_ [V]	*J*_sc_ [mA cm^−2^]	FF [%]	PCE [%]
PSK	1.11 ± 0.01	23.07 ± 0.37	71.1 ± 1.6	18.2 ± 0.6
4‐CN/PSK	1.12 ± 0.01	23.88 ± 0.26	71.3 ± 1.4	18.9 ± 0.5
PSK(10%CN)	1.12 ± 0.01	24.08 ± 0.24	71.6 ± 1.1	19.3 ± 0.4
4‐CN/PSK(10%CN)	1.13 ± 0.01	24.35 ± 0.16	73.0 ± 1.2	20.1 ± 0.5

Apart from the enhancement of photovoltaic performance, the device stability is another critical factor for planar PSCs.^[^ ^67^, ^68^ ^]^ It has been previously reported that multiple types of defects, such as vacancies of Pb^2+^ or I^−^, grain boundaries and dangling bonds, are more likely to form at the interfaces between perovskite film and its adjacent layers than the bulk perovskite.^[^ ^69^, ^70^ ^]^ The lattice in these positions is extremely fragile and effortlessly destroyed by moisture and heat, thus the decomposition of perovskite film generally begins at the interface.^[^ ^71^, ^72^ ^]^ As shown in Figure S27, Supporting Information, the moisture resistance of perovskite film has been reinforced by CN top modification. Except for passivating the defects at the surface, the incorporation of Cs^+^ and Br^−^ with smaller radii in the lattice also strengthens the ionic and covalent bonding of perovskite.^[^ ^23^ ^]^ Therefore, these two features slow down the moisture ingression kinetics in perovskite film, leading to superior moisture stability. Moreover, the long‐term stability test of PSCs is performed in atmosphere at a humidity of 60% ± 5%. As shown in Figure S28, Supporting Information, the PCE of 4‐CN/PSK(10%CN) based PSC still maintains above 90% after 36 days, whereas the pristine PSK based PSC dramatically drops below 80%. This further confirms that the CN bilateral interface modification can reduce the defect density inside the device and increase its tolerance to moisture.

## 3 Conclusion

In summary, we have demonstrated a simple and effective strategy to ameliorate the perovskite active layers through bilateral interface modification using room‐temperature synthesized CN. Through CN bottom modification, CN is uniformly introduced on the surface of SnO_2_ ETL, where CN based seed crystal can initiate the seed‐mediated growth of perovskite to reduce the ionic defects in perovskite films and facilitate interfacial electron transport. With CN top modification, a graded heterojunction is constructed at the upper part of perovskite film, which increases the difference between Fermi levels at the bottom and surface of perovskite film. This results in a stronger built‐in electric field which is advantageous for the separation and oriented migration of electrons and holes inside the perovskite film. Combining the merits of CN bottom and top modification, the CN bilateral interface modified MAPbI_3_‐based PSCs demonstrate an average PCE exceeding 20% and ameliorative device stability. Consequently, our work provides significant insights into the systematic utilization of perovskite nanocrystal to boost the photovoltaic performance of planar PSCs, which facilitates the advancement of perovskite based optoelectronic devices.

## Conflict of Interest

The authors declare no conflict of interest.

## Supporting information

Supporting Information

## Data Availability

Research data are not shared.
