# Supplementary material for: CsPbBr3 Nanocrystal Induced Bilateral Interface Modification for Efficient Planar Perovskite Solar Cells
Source: Adv Sci (Weinh). 2021 Sep 13;8(21):2102648. doi: 10.1002/advs.202102648 (PMC8564463; doi:10.1002/advs.202102648)
Supplement: Supplementary file 1 — Supporting Information [file ADVS-8-2102648-s001.pdf]

WILEY-VCH

© 2021 Wiley-VCH GmbH

ADVANCED SCIENCE
Open Access

Supporting Information

for *Adv. Sci.*, DOI: 10.1002/advs.202102648

$CsPbBr_3$ Nanocrystal Induced Bilateral Interface Modification for Efficient Planar Perovskite Solar Cells

Jianjun Zhang, Linxi Wang Chenhui Jiang, Bei Cheng, Tao Chen and Jiaguo Yu**

© 2021 Wiley-VCH GmbH

Supporting Information

$CsPbBr_3$ Nanocrystal Induced Bilateral Interface Modification for Efficient Planar Perovskite Solar Cells

Jianjun Zhang, Linxi Wang Chenhui Jiang, Bei Cheng, Tao Chen and Jiaguo Yu**

Experimental Section

Materials: The $PbBr_2$ (purity≥99%) and Cs_2CO_3 (purity≥99%) were purchased from Aladdin Reagents Co., Ltd. The PbI_2 (purity≥99.99%), CH_3NH_3I (MAI) (purity≥99.5%), 2,2′,7,7′-tetrakis[N,N-di(4-methoxyphenyl)amino]-9,9′-spirobifluorene (purity≥99.8%) and Li-bis (trifluoromethanesulfonyl) imide (Li-TFSI) were obtained from Xi'an Polymer Light Technology Corp. N,N-dimethylformamide (DMF) and dimethyl sulfoxide (DMSO) (purity≥99.8%) were purchased from J&K Scientific. The 4-tert-butylpyridine (TBP) was purchased from Acros Organics. Other reagents were obtained from Sinopharm Chemical Reagent Co., Ltd. All reagents were used as received without further purification.

$CsPbBr_3$ Nanocrystal (CN) Synthesis: CN was prepared through a previously reported room-temperature synthesized procedure.[S1] Briefly, Cs precursor (3.6 M Cs^+) was prepared by dissolving 2.934 g of Cs_2CO_3 in 5 mL of propionic acid. Pb precursor (0.5 M Pb^{2+}) was synthesized by dissolving 0.275 g of $PbBr_2$ in 500 μL of propionic acid, 500 μL of 2-propanol and 500 μL of butylamine. Then 6 mL of n-hexane, 3 mL of isopropanol and 20 μL of Cs precursor were mixed in air at room temperature. 300 μL of Pb precursor was quickly injected into the aforementioned mixture to obtain the CN suspension. Finally, the CN suspension was centrifuged and redispersed in 20 mL of toluene for further use.

Planar PSC Fabrication: Fluorine-doped tin oxide (FTO) glass substrates were sonicated with deionized water, acetone, and ethanol for 30 min in sequence. UV-ozone treatment was conducted for 15 min to remove the residual organics and increase the hydrophilicity of FTO. SnO_2 electron-transport layer (ETL) was prepared by spin-coating 100 μL of SnO_2 precursor solution (1.015 g of $SnCl_2{\cdot}2H_2O$ and 0.342 g of thiourea dissolved in 30 mL of deionized water, and the solution was stirred vigorously for 24 hours to obtain the transparent yellow precursor solution) onto the cleaned FTO substrate, followed by annealing at 200 °C for 1 h. Then, the deposition of the perovskite layer and spiro-OMeTAD hole-transport layer (HTL) was conducted in an argon-filled glovebox. The 1.3 M of perovskite precursor was prepared by dissolving PbI_2 and CH_3NH_3I in DMF and DMSO (volume ratio 4:1). The perovskite layer was prepared by spin-coating 40 μL of perovskite precursor onto the SnO_2 layer at 4000 rpm for 30 s, during which 200 μL of toluene was dropped on the spinning substrate during the first 10 s. Then, the film was annealed at 100 °C for 10 min. Spiro-OMeTAD HTL was prepared by spin-coating 40 μL of spiro-OMeTAD solution (74 mg of spiro-OMeTAD, 18.2 mg of Li-TFSI, and 28.8 μL of TBP dissolved in a mixture of 35 μL of acetonitrile and 1 of mL chlorobenzene) on the perovskite layer at 3000 rpm for 30 s. Finally, 50 nm-thick Au electrodes were thermally evaporated onto the hole-transport layer. The active area of the PSC was 0.09 cm^2.

CN modification: (1) As for CN bottom modification, prior to the deposition of perovskite layer, 100 μL of prepared toluene with dispersed CN was spinning-coated onto the SnO_2 ETL for 2 times, 4 times and 6 times. The SnO_2 films with different times of CN bottom modification were marked as SnO_2/2-CN, SnO_2/4-CN and SnO_2/6-CN. The perovskite films deposited on the CN modified SnO_2 films were labeled as 2-CN/PSK, 4-CN/PSK and 6-CN/PSK, respectively; (2) For CN surface modification, during the deposition of the perovskite film, the previously prepared CN/TL suspension was diluted to 5%, 10% and 20% of the original concentration and used as

the antisolvent to deposit the perovskite film. The prepared perovskite films are marked as PSK(5%CN), PSK(10%CN) and PSK(20%CN); (3) As for the CN bilateral surface modification, the optimal conditions for bottom modification and surface modification are utilized to fabricate PSCs.

Characterizations: The morphology of the perovskite films were tested on a JSM-7500F field emission scanning electron microscope (SEM, JEOL, Japan) and multimode 8 atomic force microscope (AFM, Bruker, USA) in ScanAnalyst mode. The transmission electron microscopy (TEM) images of CN were conducted on a scanning transmission electron microscope (STEM, Titan G2 60-300, FEI, USA). X-ray diffraction (XRD) were performed on an X-ray diffractometer (XRD-6100, λ = 0.15 nm, Shimadzu, Japan). Glancing-angle XRD tests were recorded on an X-ray diffractometer (Empyrean, PANalytical, Netherlands). The contact angle test was conducted on contact angle meter (Theta Lite, Attension, Finland). UV–vis absorption spectra were obtained on a UV–visible spectrometer (UV2600, Shimadzu, Japan). Steady-state photoluminescence (PL) spectra were carried out on a fluorescence spectrophotometer (F-7000, Hitachi, Japan) with 512 nm excitation wavelength. Time-resolved transient photoluminescence (TRPL) decay curves were tested on a FLS1000 fluorescence lifetime spectrophotometer (Edinburgh Instruments, U.K.) with 450 nm excitation wavelength. The ultrafast transient absorption (TA) measurements were performed on a pump–probe system (Helios, Ultrafast System) with the maximum time delay of ~8 ns using a motorized optical delay line under ambient conditions. The pump pulses at 400 nm (~20 μW average power at the sample) were delivered by an ultrafast optical parametric amplifier (OPera Solo) excited by a regenerative amplifier (Coherent Astrella, 800 nm, 35 fs, 5 mJ, 1 kHz), seeded with a mode-locked Ti:sapphire oscillator (Coherent Vitara, 800 nm, 80 MHz) and pumped with a LBO laser (Coherent Evolution-50C, 1 kHz system). Ultroviolet photoelectron spectra (UPS) were measured on Thermo ESCALAB 250. Kelvin probe force microscopy (KPFM) images were tested on the scanning probe microscope (SPM9700HT,

Shimadzu, Japan). The iron plate is utilized to calibrate the zero-potential point of the probe and the test structure is the glass/perovskite film. Contact potential difference (CPD) was conducted on a kelvin probe apparatus (Instytut Fotonowy, Poland). The PSK, PSK(10%CN) and CN are deposited on the ITO substrates to ensure that the test samples and the gold probe are electrically connected via internal circuitry. Subsequently, the CPD between the surface of samples and the standard gold probe can be acquired via modulating their surface charge balance at extremely close distance. The current density–voltage (*J*–*V*) of PSC devices was performed on an electrochemical work station (CHI660C, Chenhua Instrument Corp., China) under simulated 100 mW cm^{-2} intensity (AM 1.5G, 1 Sun) by a solar simulator (91160, Newport Corp., Irvine, CA, USA). The dark *J*–*V* and electrochemical impedance spectra (EIS) were collected under dark condition over the frequency range of 0.01–10^5 Hz. The SCLC test was carried out on Keithley 2450. The incident photon-to-current conversion efficiency (IPCE) was obtained on Newport's QE/IPCE Measurement Kit with monochromatic light from a 300 W Xe lamp (Newport, model no. 6258).

Statistical Analysis: All data presented represent the mean values of multiple collected scans. The errors given for measurements were based on standard deviation on these average values. All XRD data were original without background subtraction. TEM data was analyzed by Digital Micrograph. AFM data was processed by NanoScope Analysis. TA data was analyzed by Surface Xplorer. Other data was processed by Origin. Specific details for all methods are discussed in the Characterization Section.

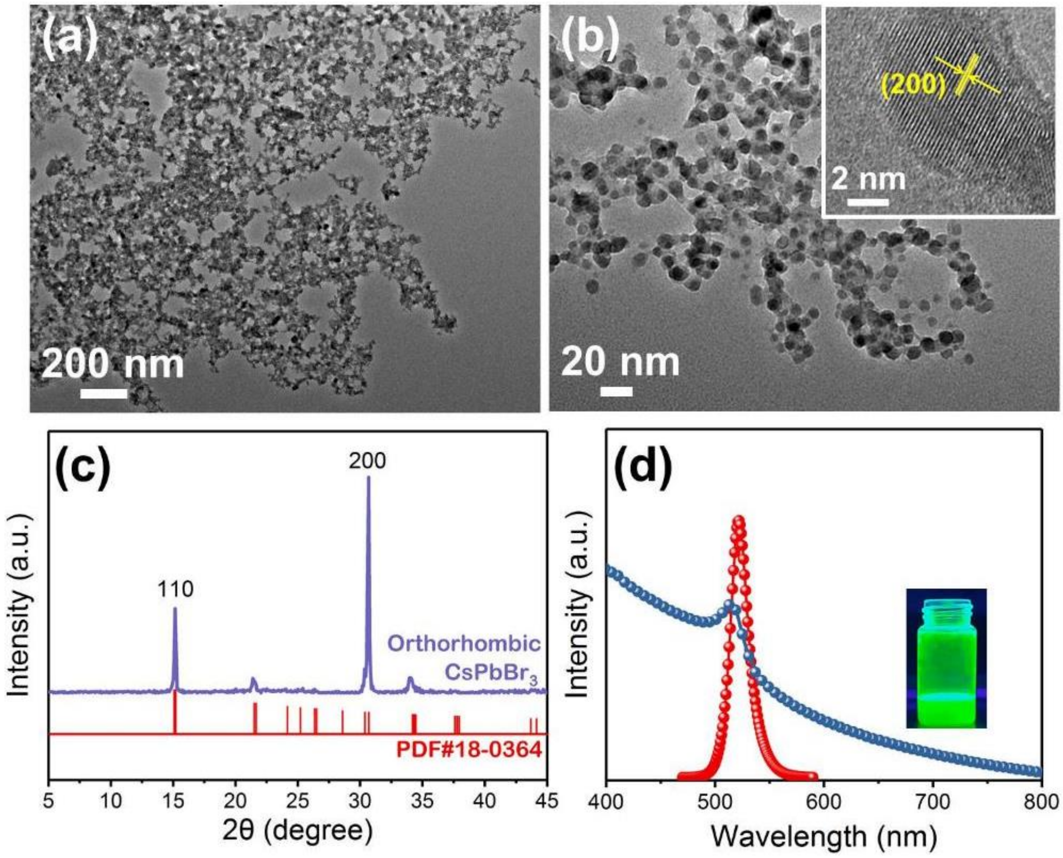

Figure S1. a–b) TEM image and high-resolution TEM images of room-temperature synthesized CN. c) XRD pattern of CN. d) UV-vis absorption and steady-state PL spectra of CN. The inset is the optical photograph of CN solution under 365 nm light.

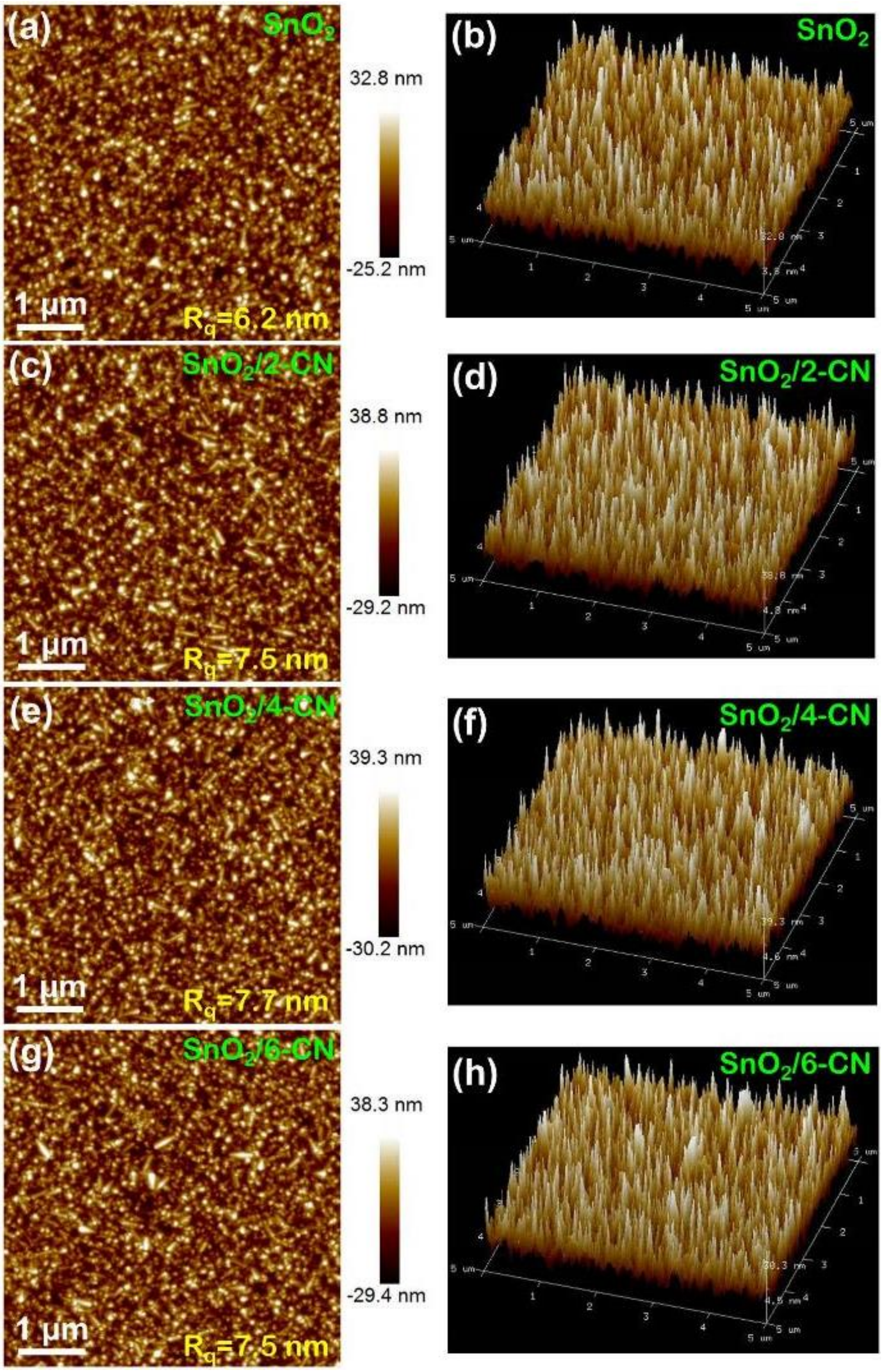

Figure S2. AFM images and 3D AFM images of a–b) SnO_2 film, c–d) SnO_2/2-CN film, e–f) SnO_2/4-CN film and g–h) SnO_2/6-CN film in the region of 5 µm × 5 µm.

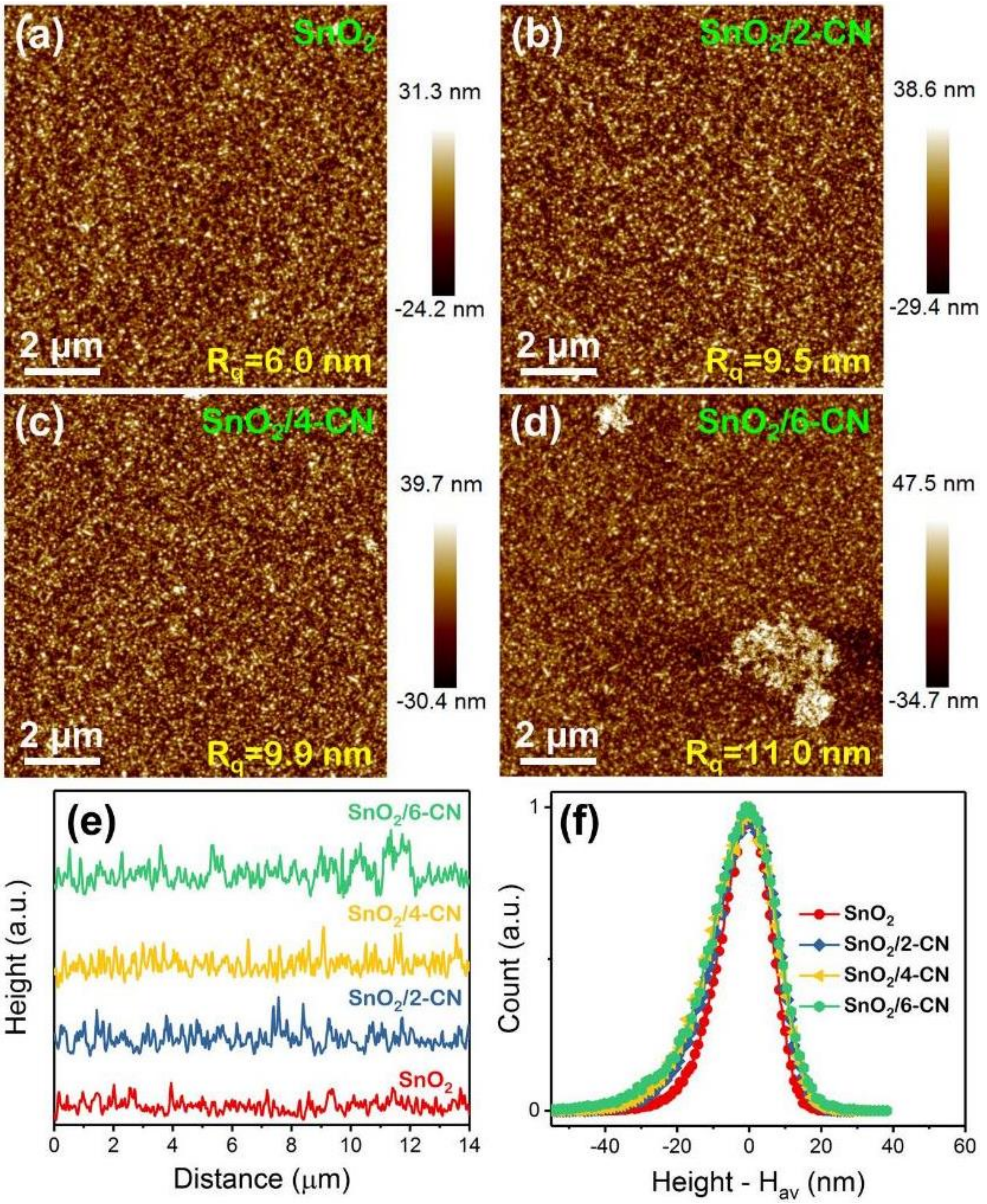

Figure S3. a–d) AFM images, e) line segments, f) depth distribution with the average height (H_{av}) of SnO_2 films without and with different times of CN modification in the region of 10 µm × 10 µm.

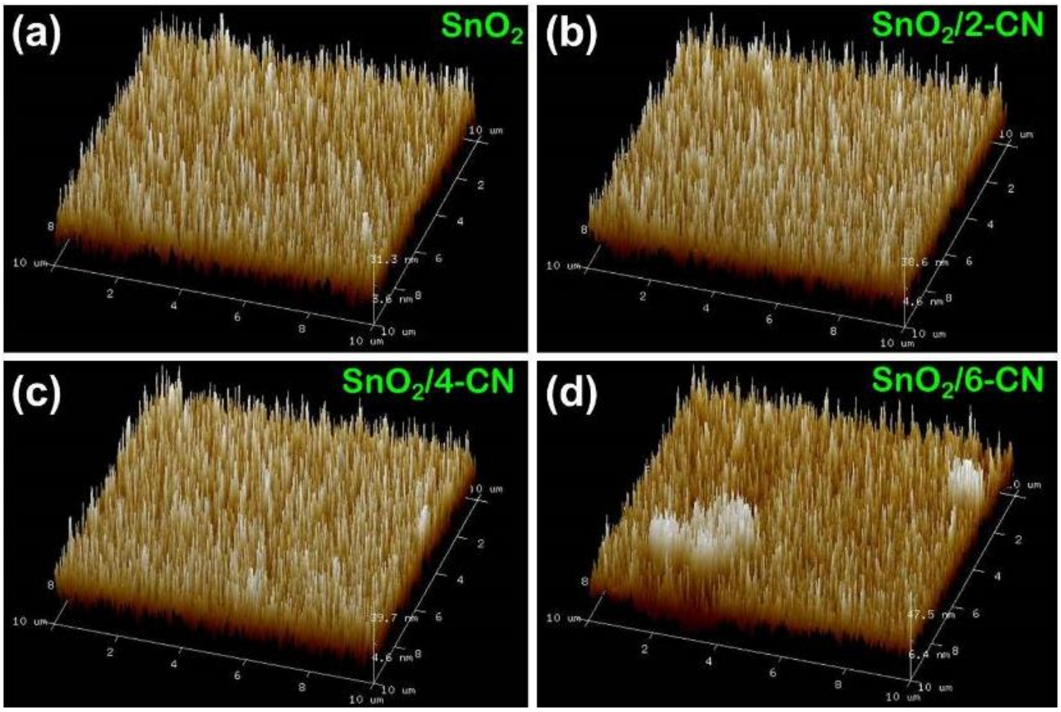

Figure S4. a–d) 3D AFM images of SnO_2 films without and with different times of CN modification in the region of 10 µm × 10 µm.

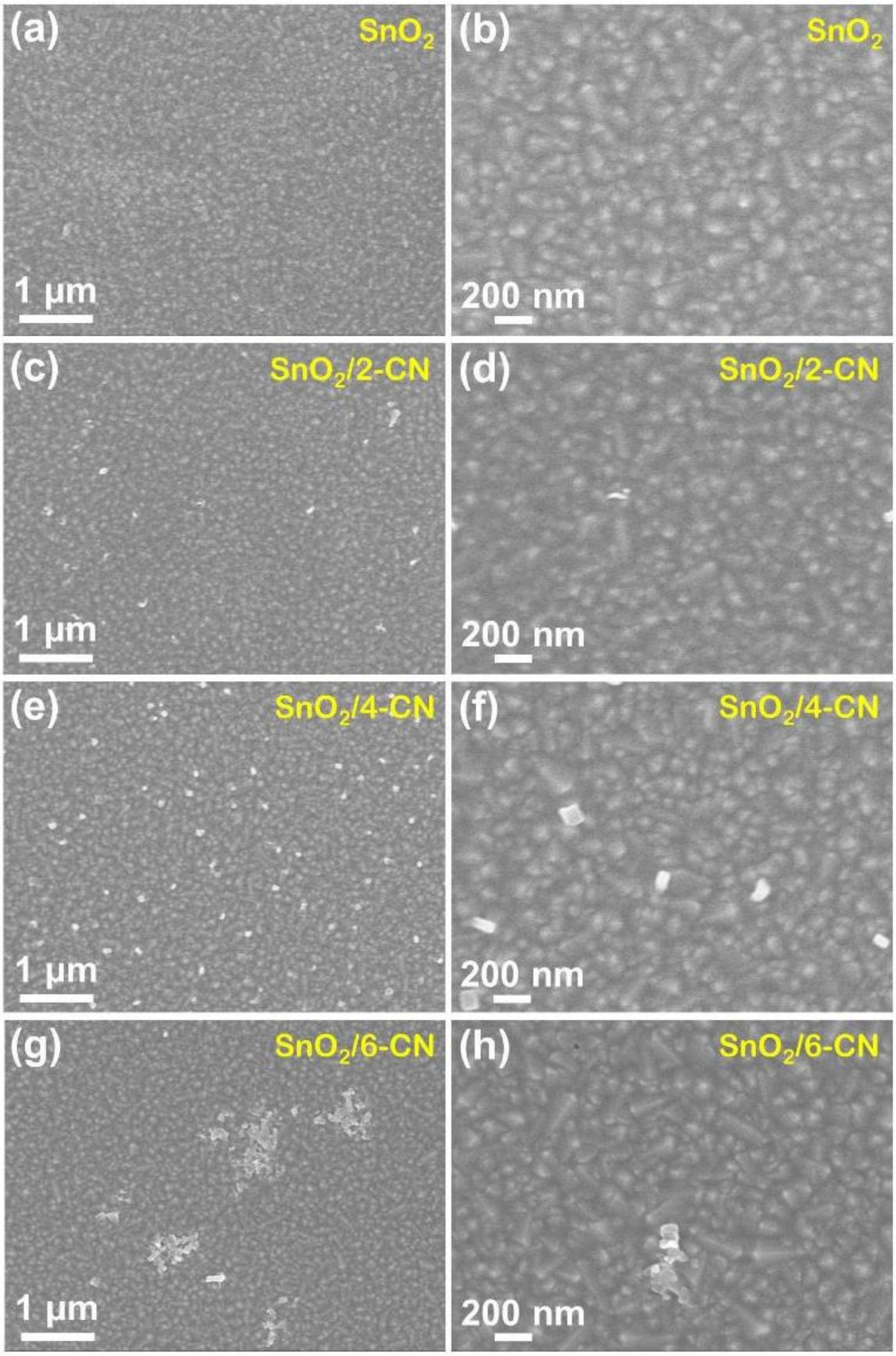

Figure S5. Top-view SEM images of (a–b) SnO_2 film, (c–d) SnO_2/2-CN film, (e–f) SnO_2/4-CN film and (g–h) SnO_2/6-CN film.

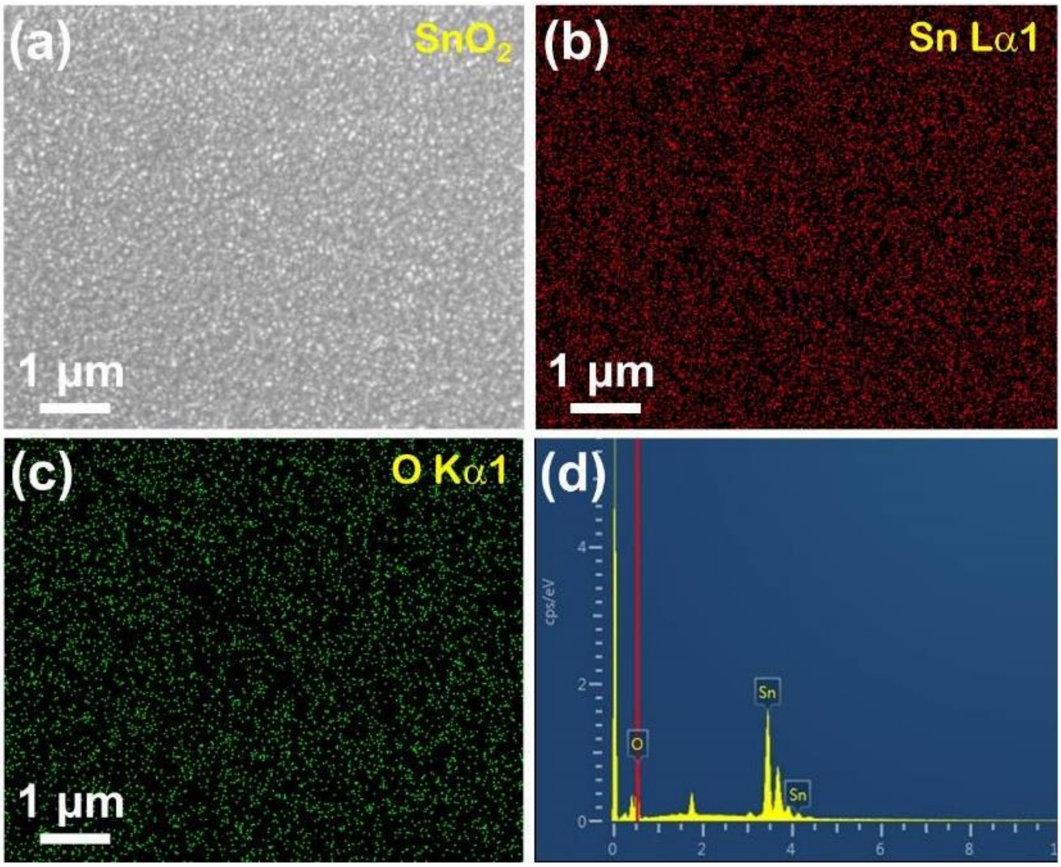

Figure S6. a) SEM image, b–c) EDS mapping and d) EDS spectrum of SnO_2 film.

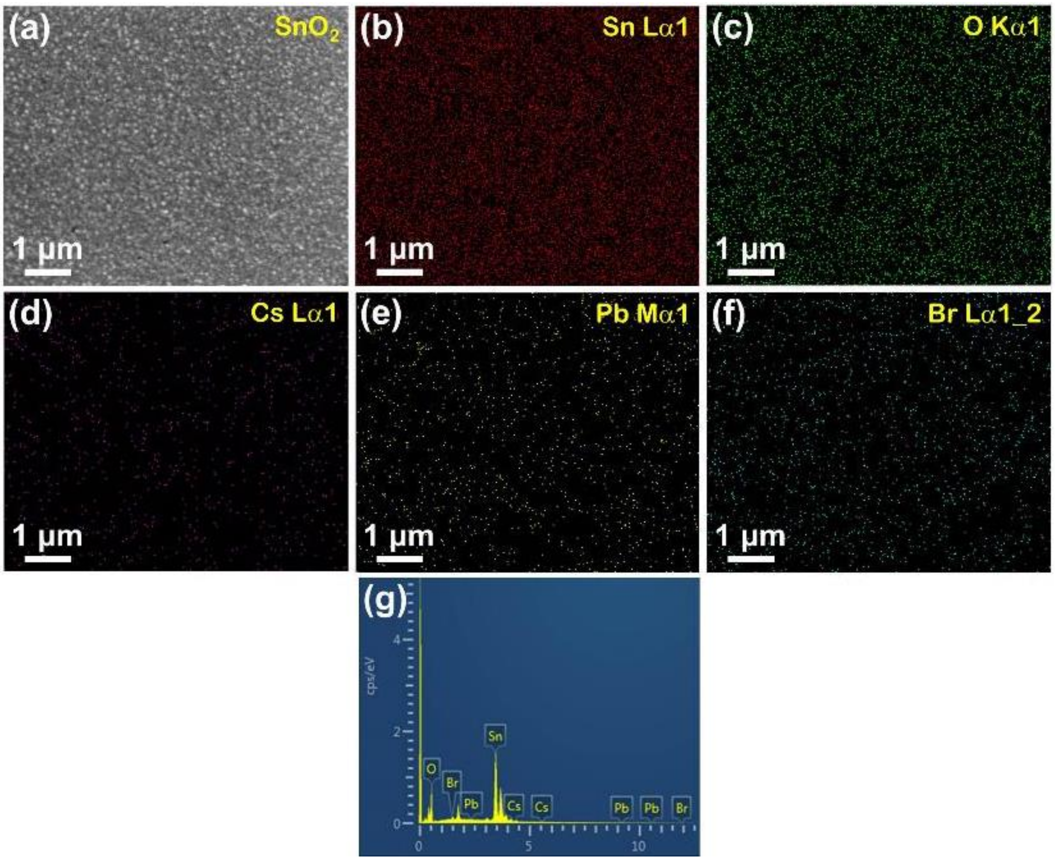

Figure S7. a) SEM image, b–f) EDS mapping and g) EDS spectrum of SnO_2/4-CN film.

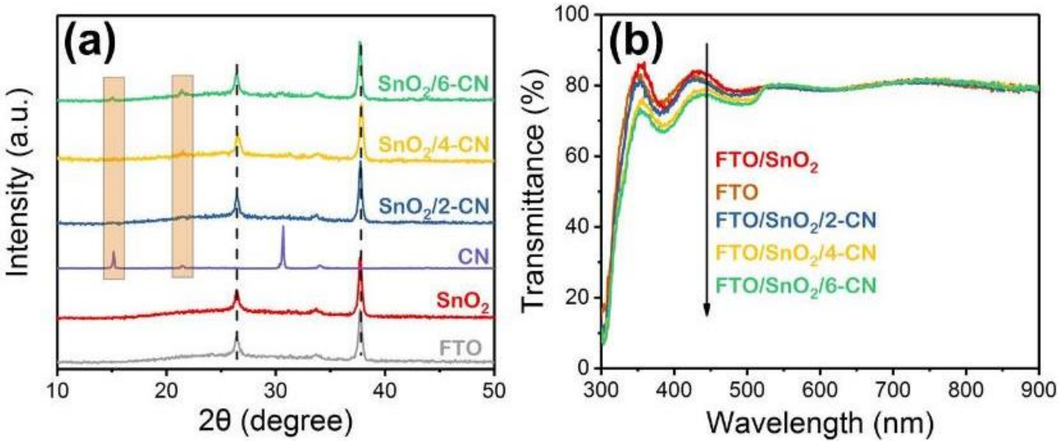

Figure S8. a) XRD patterns of FTO, CN and FTO/SnO_2 substrates without and with different times of CN modification. b) Transmittance spectra of FTO substrate and FTO/SnO_2 substrates without and with various times of CN modification.

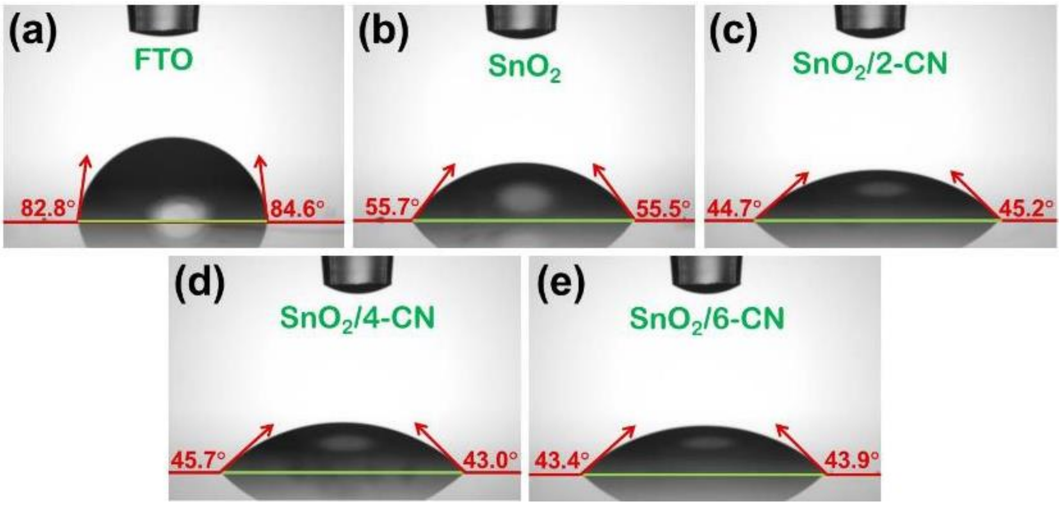

Figure S9. The contact angles of water droplets on a) FTO, b) SnO_2 film, c) SnO_2/2-CN film, d) SnO_2/4-CN film and e) SnO_2/6-CN film.

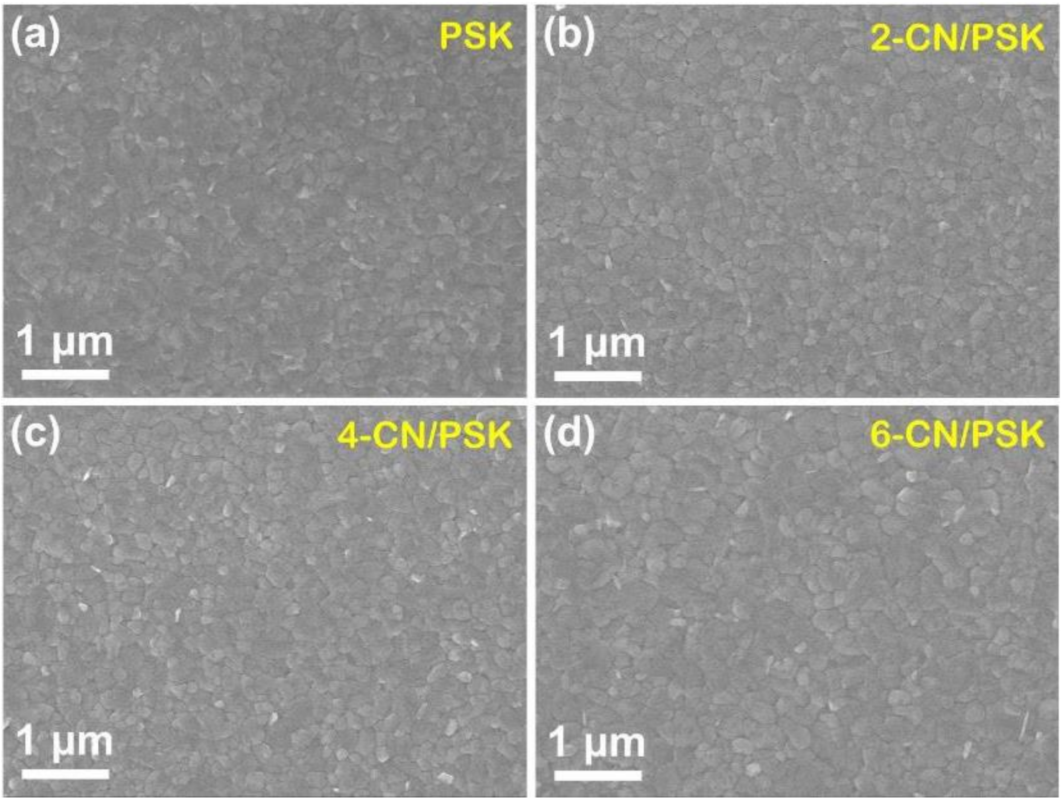

Figure S10. Top-view SEM images of perovskite films deposited on a) SnO_2 film, b) SnO_2/2-CN film, c) SnO_2/4-CN film and d) SnO_2/6-CN film.

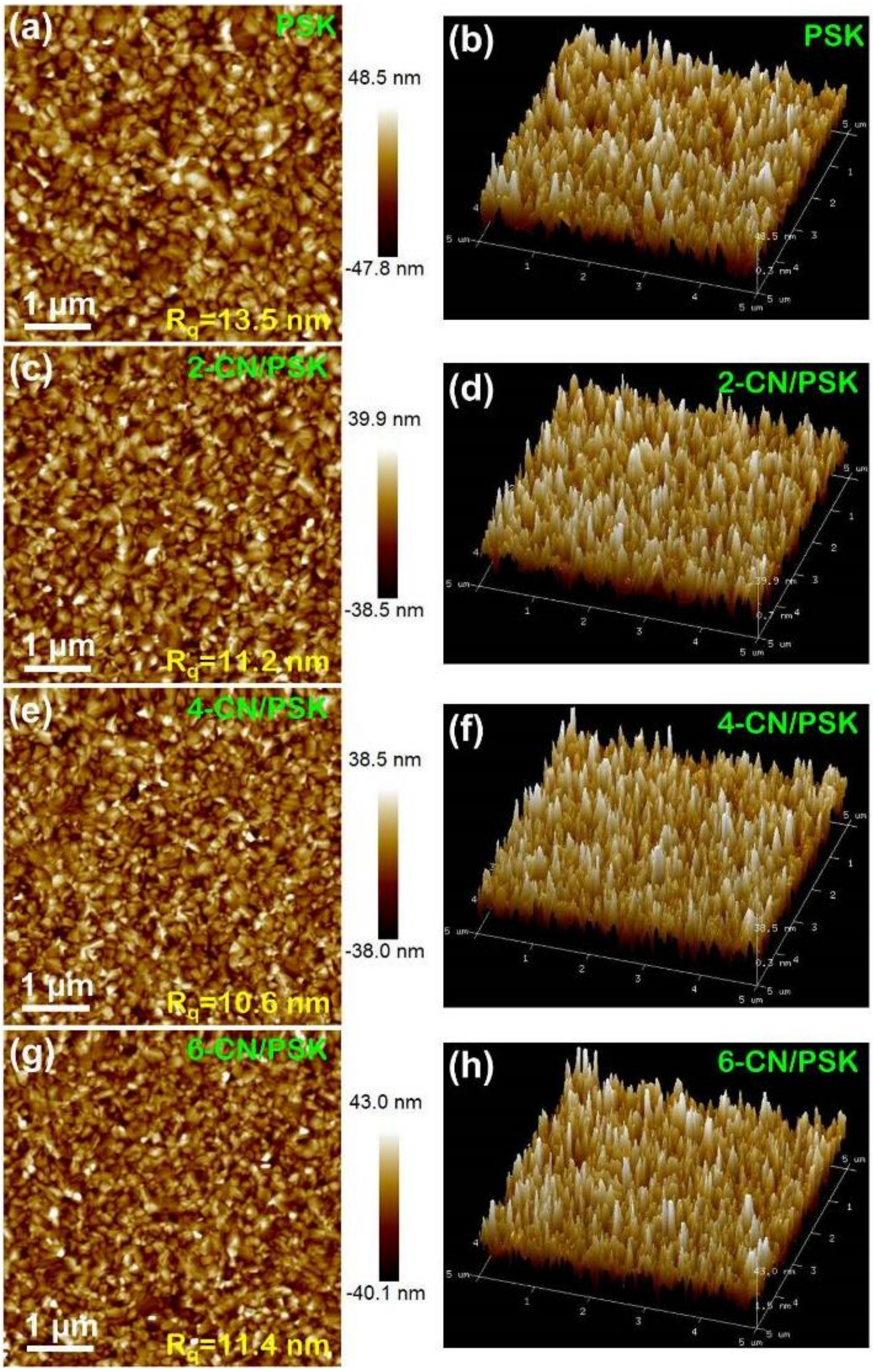

Figure S11. AFM images and 3D AFM images of perovskite films deposited on a,b) SnO_2 film, c,d) SnO_2/2-CN film, e,f) SnO_2/4-CN film and g,h) SnO_2/6-CN film in the region of 5 μm × 5 μm.

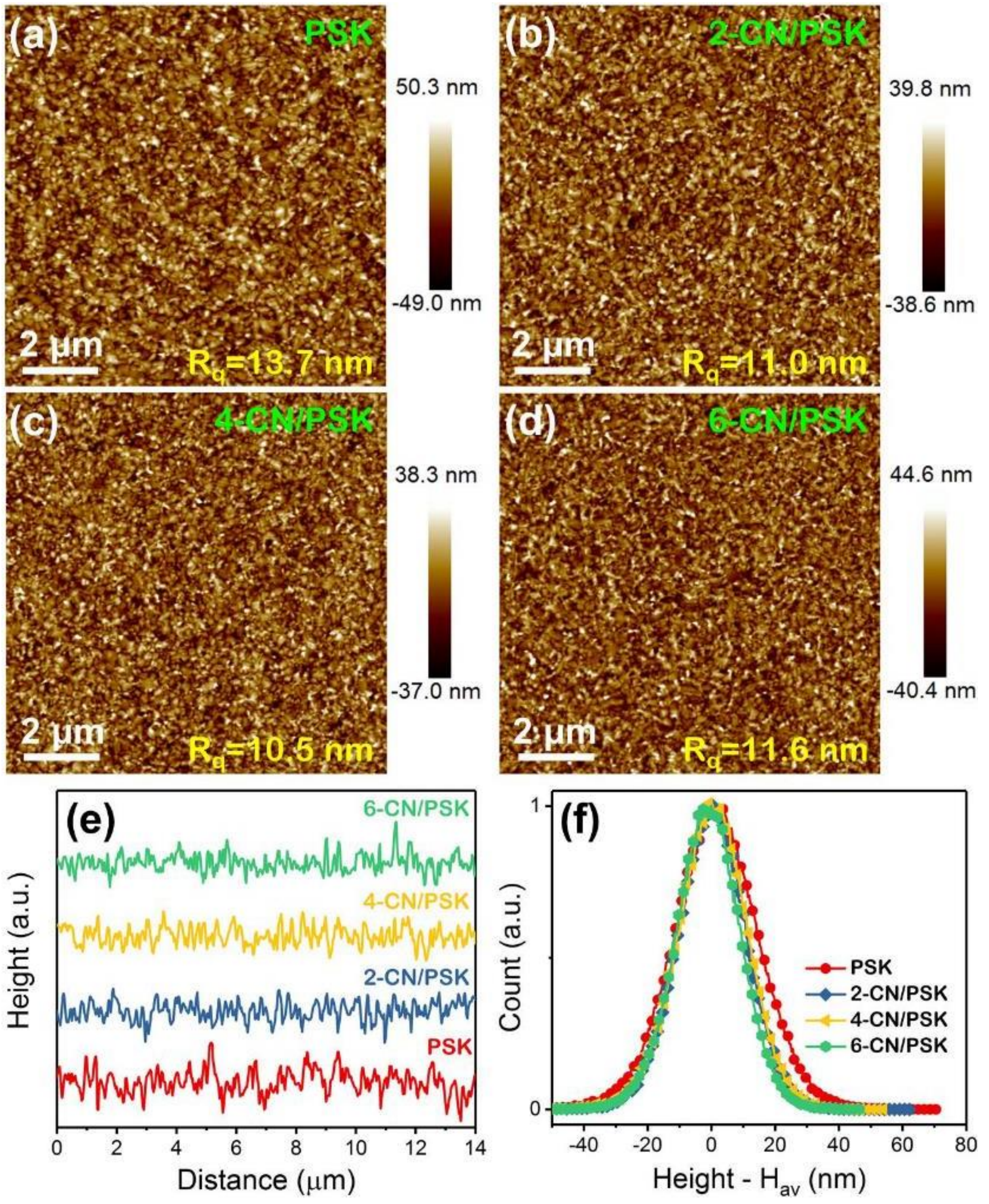

Figure S12. a–d) AFM images, e) line segments and f) depth distribution with the average height (H_{av}) of perovskite film deposited on SnO_2 film without and with different times of CN modification in the region of 10 µm × 10 µm.

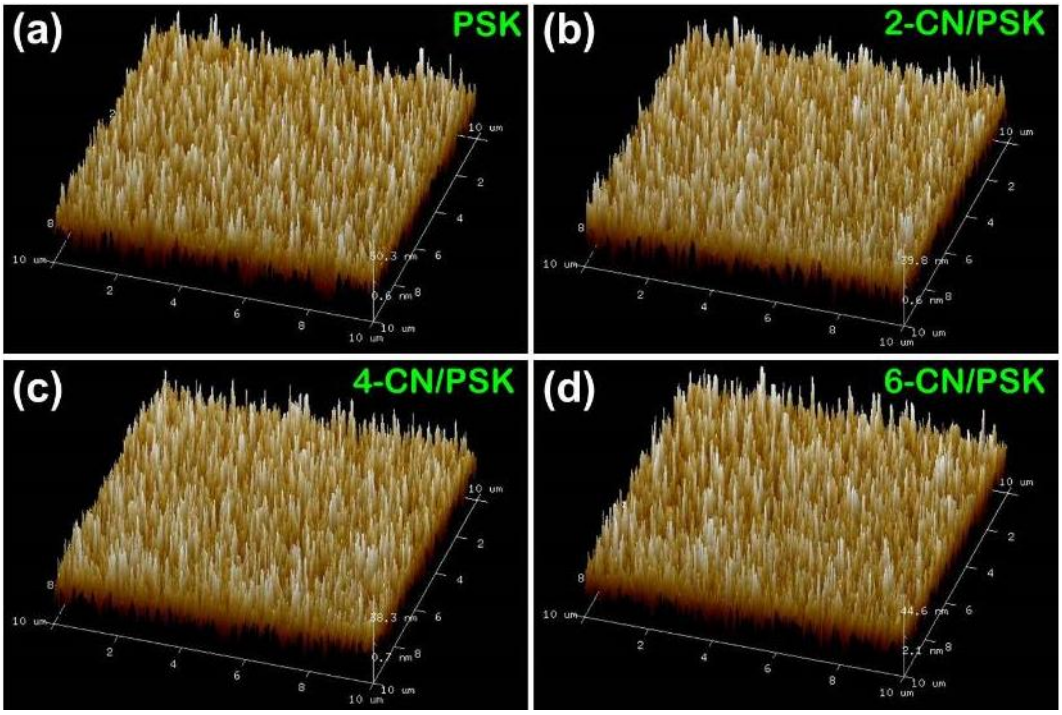

Figure S13. a–d) 3D AFM images of perovskite films deposited on SnO_2 film without and with different times of CN modification in the region of 10 μm × 10 μm.

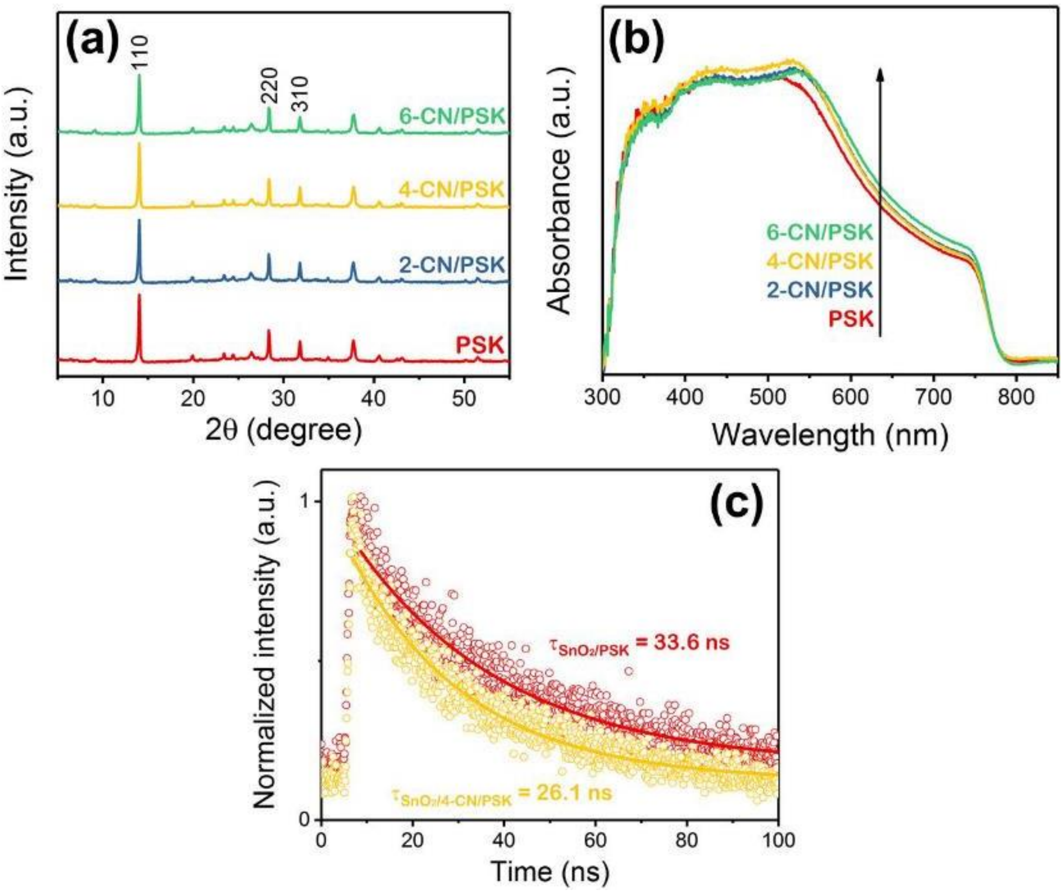

Figure S14. a) XRD patterns and b) UV–vis absorption spectra of perovskite films deposited on SnO_2 film without and with various times of CN modification. c) TRPL curves of perovskite films deposited on individual SnO_2 film and SnO_2/4-CN film.

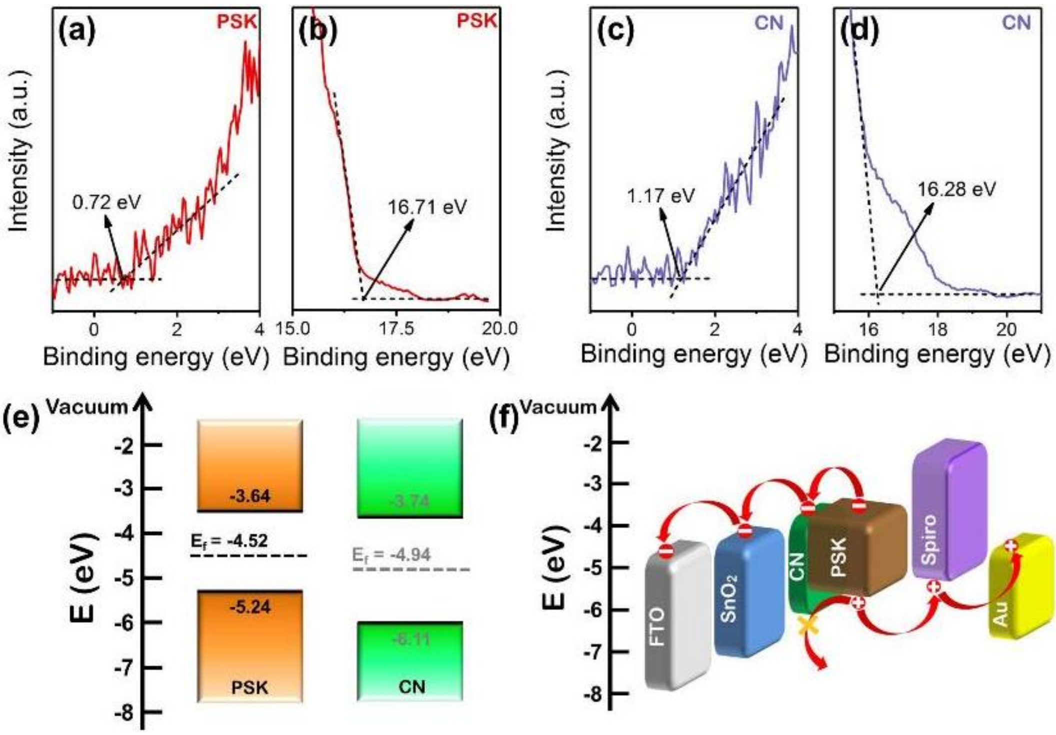

Figure S15. UPS spectra of a–b) PSK and c–d) CN. e) The position distributions of conduction band (CB), valence band (VB) and Fermi level (E_f) for PSK and CN. f) Schematic of charge transport process in PSCs with CN bottom modification.

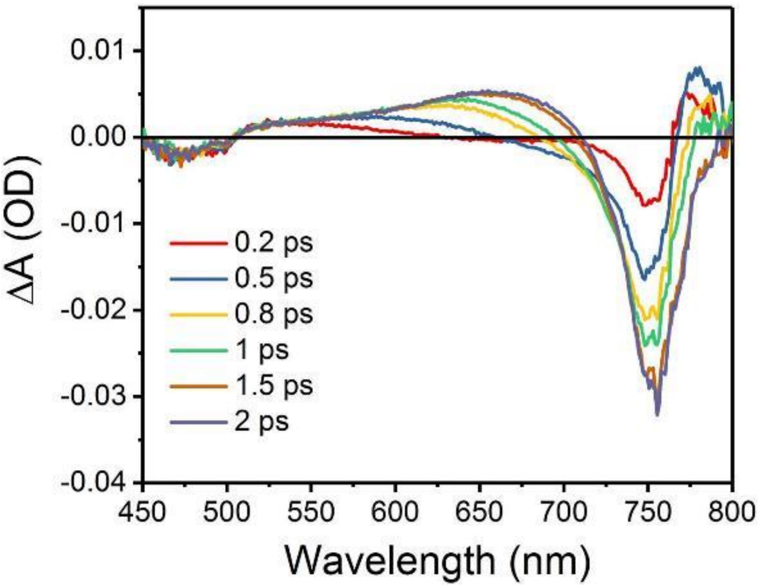

Figure S16. Transient TA spectra acquired at probe delays within 2 ps with a 400 nm laser pulse excitation for $FTO/SnO_2/PSK$.

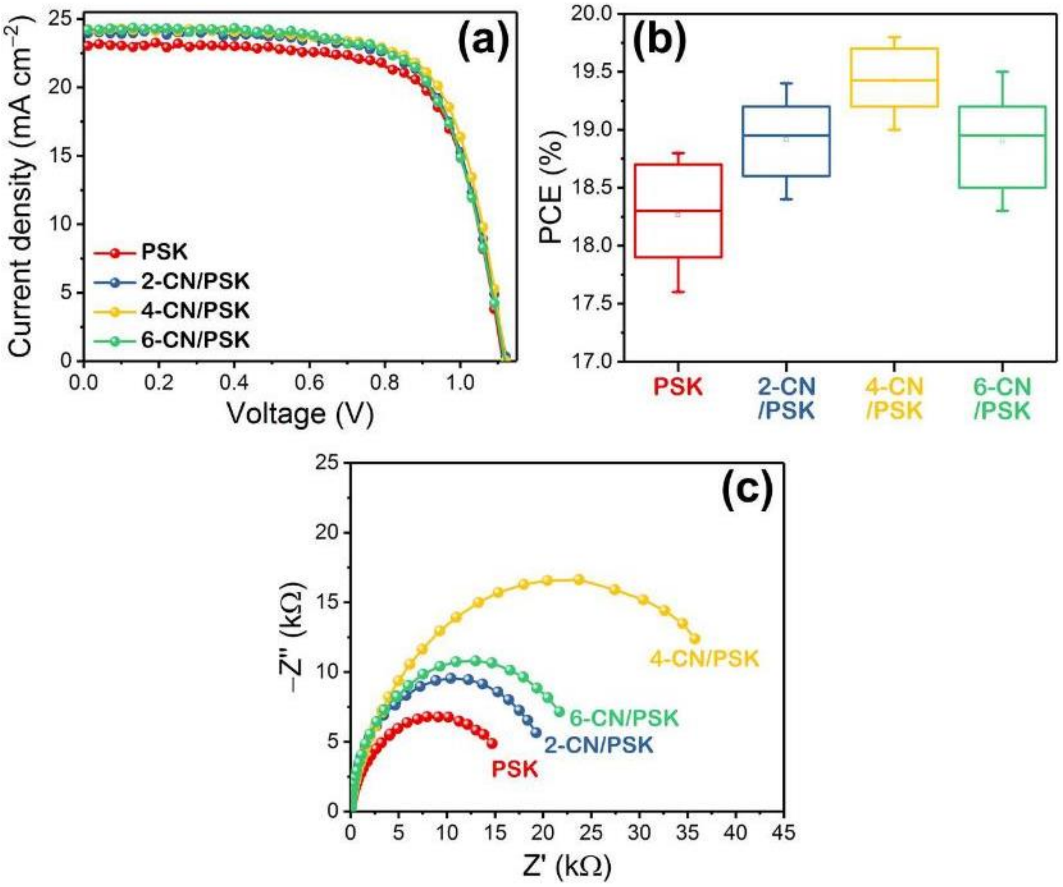

Figure S17. a) *J*–*V* curves under simulated AM 1.5 illumination, b) PCE distributions (25 devices are fabricated for each sample) and c) EIS plots recorded under dark condition for PSCs based on different times of CN bottom modification.

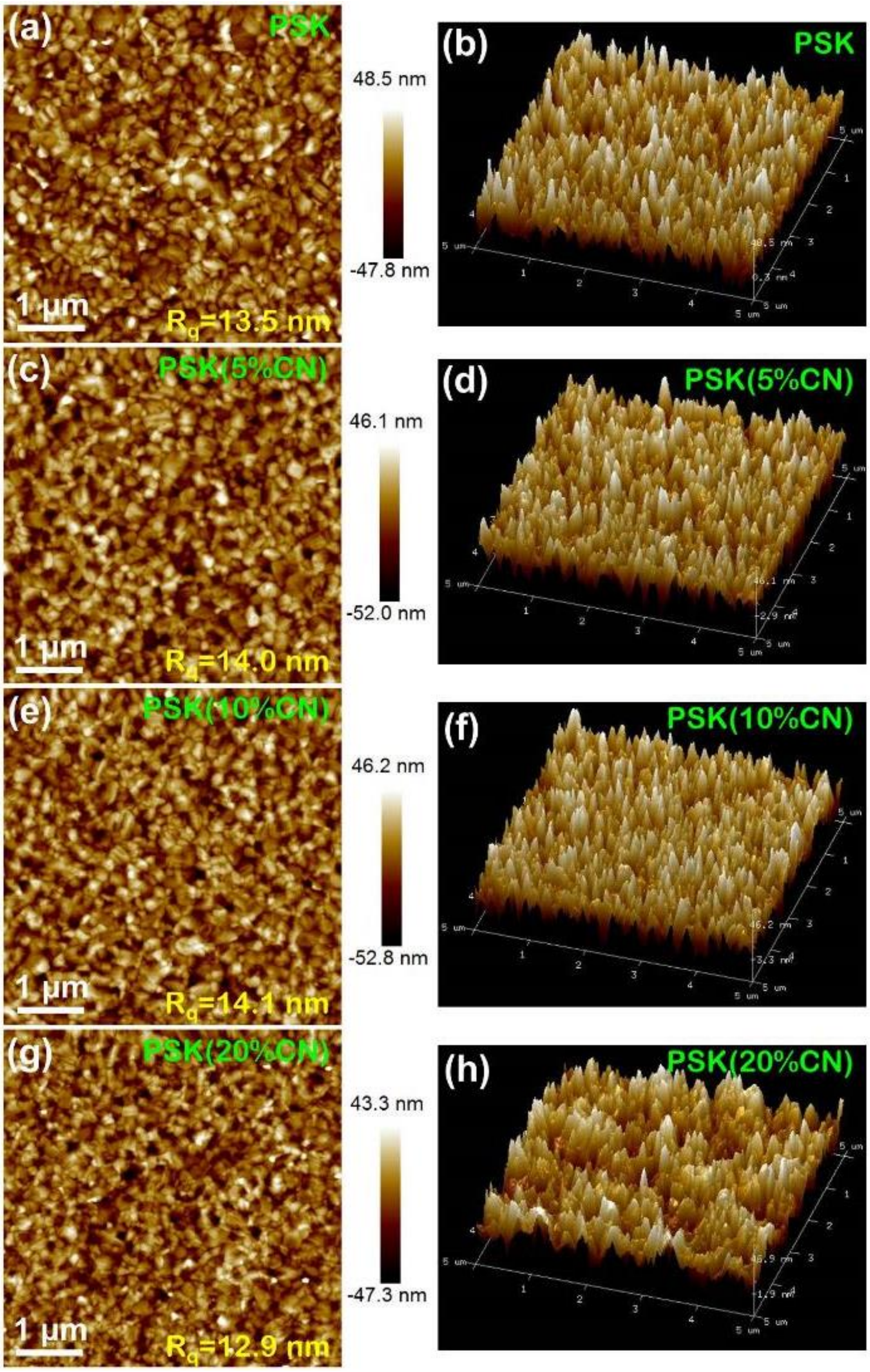

Figure S18. a–h) AFM images and 3D AFM images of perovskite films prepared via toluene containing different concentrations of CN in the region of 5 µm × 5 µm.

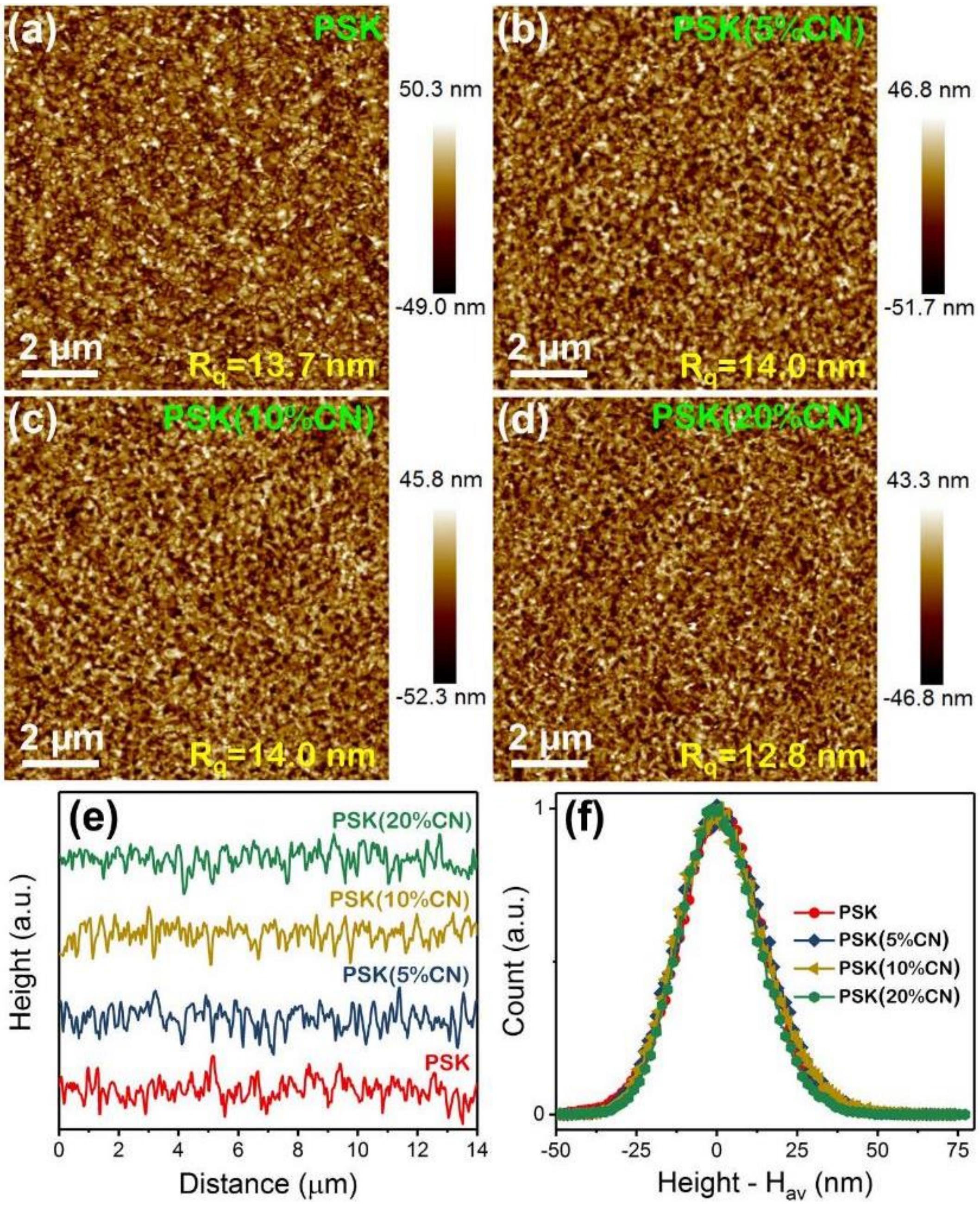

Figure S19. a–d) AFM images, e) Line segments and f) depth distribution with the average height (H_{av}) of perovskite films prepared through toluene containing different concentrations of CN in the region of 10 µm × 10 µm.

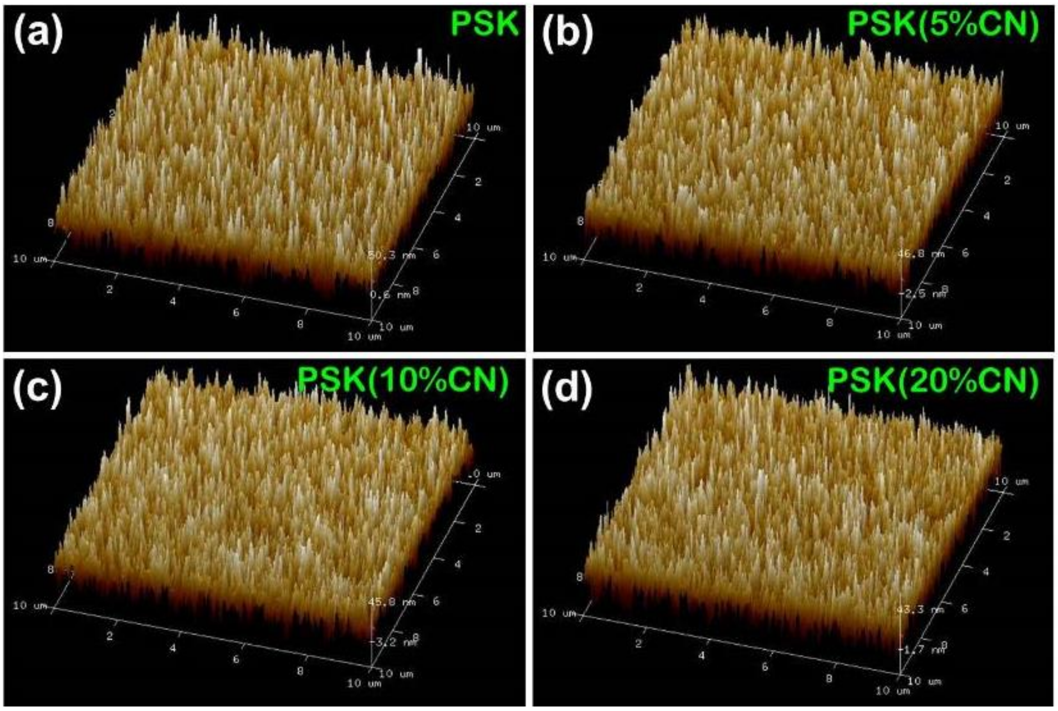

Figure S20. a–d) 3D AFM images of perovskite films prepared through antisolvent containing different concentrations of CN in the region of 10 μm × 10 μm.

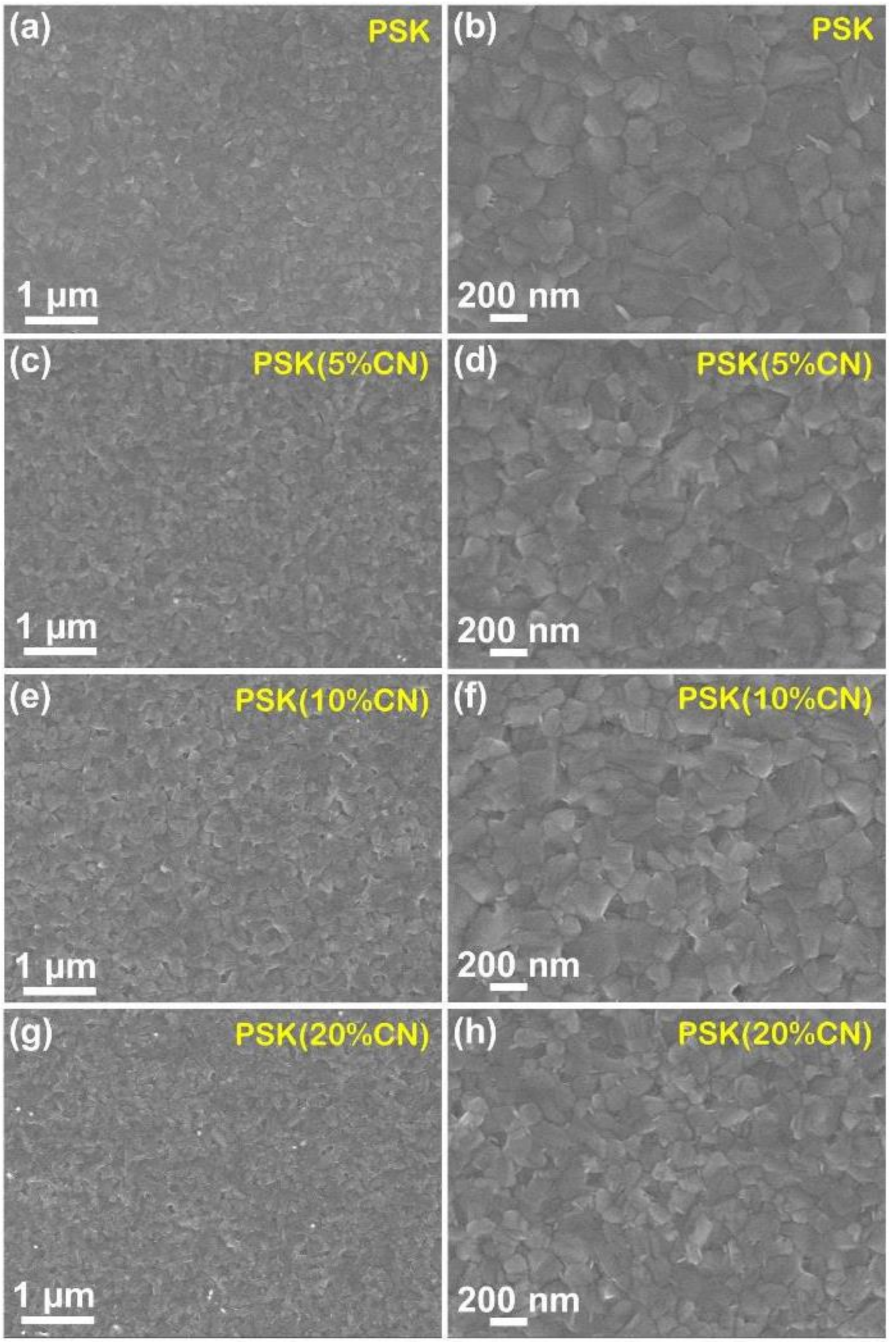

Figure S21. Top-view SEM images of a–b) PSK, c–d) PSK(5%CN), e–f) PSK(10%CN) and g–h) PSK(20%CN).

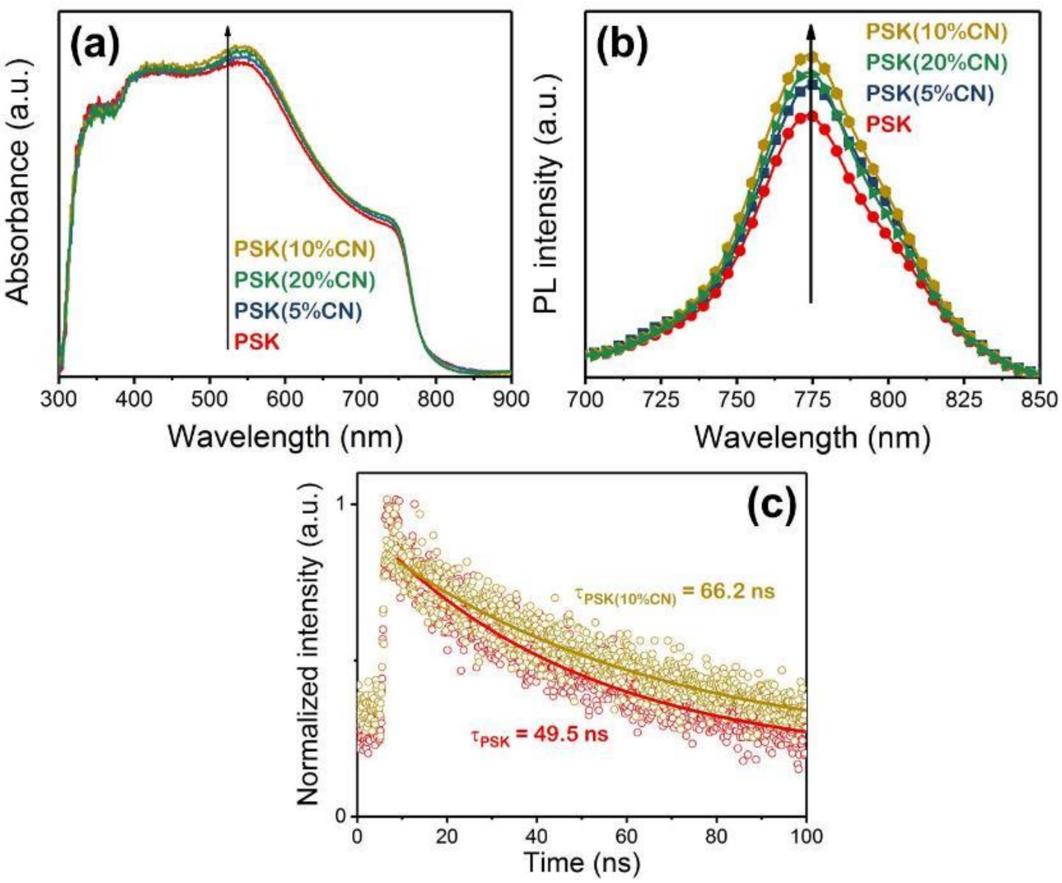

Figure S22. a) UV–vis absorption spectra and b) steady-state PL spectra of perovskite films synthesized through antisolvent containing different concentrations of CN. c) TRPL spectra of PSK film and PSK(10%CN) film.

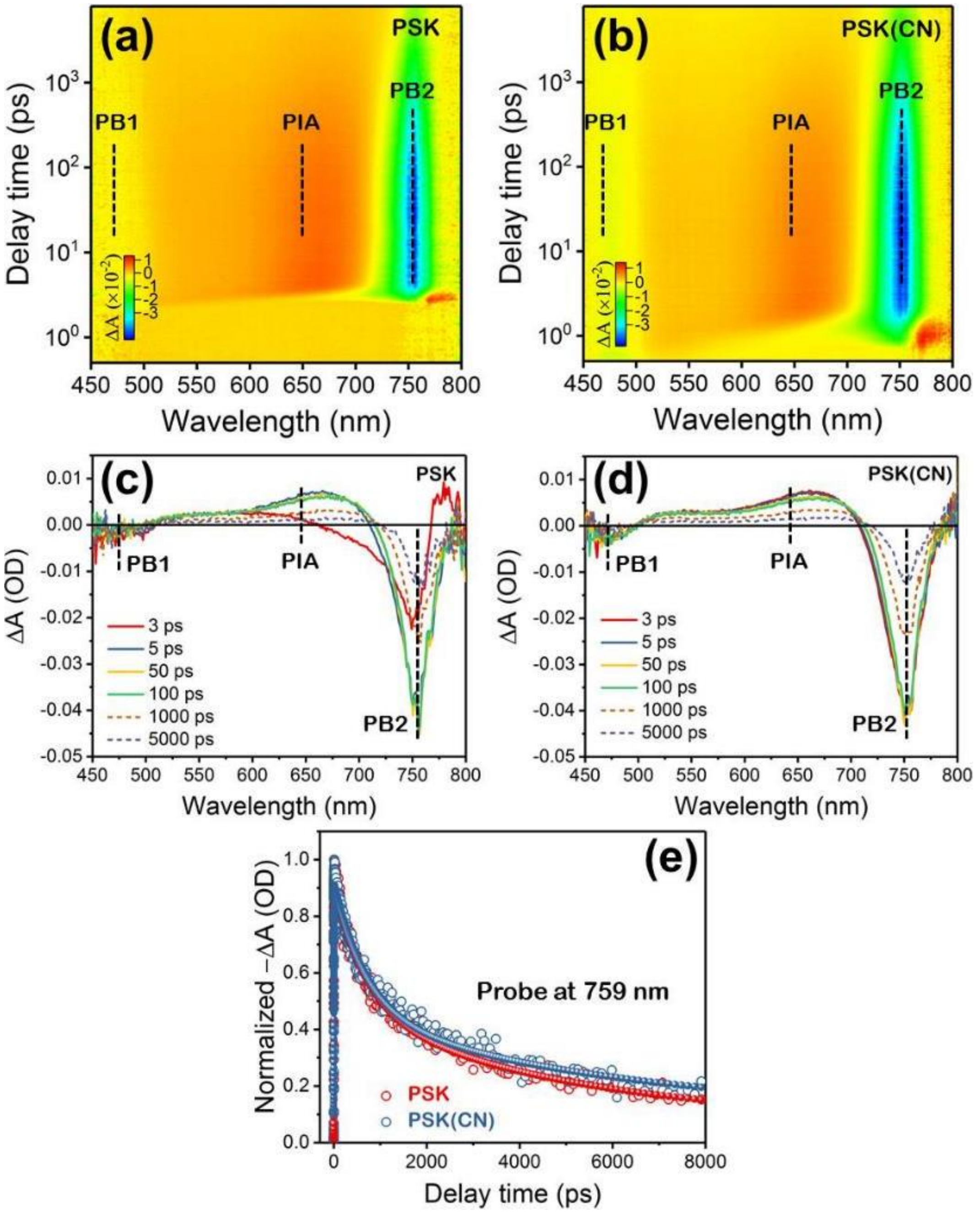

Figure S23. a,b) The pseudocolor plots, c,d) transient TA spectra of PSK and PSK(CN) excited at 400 nm, and e) corresponding normalized decay kinetic curves at 759 nm.

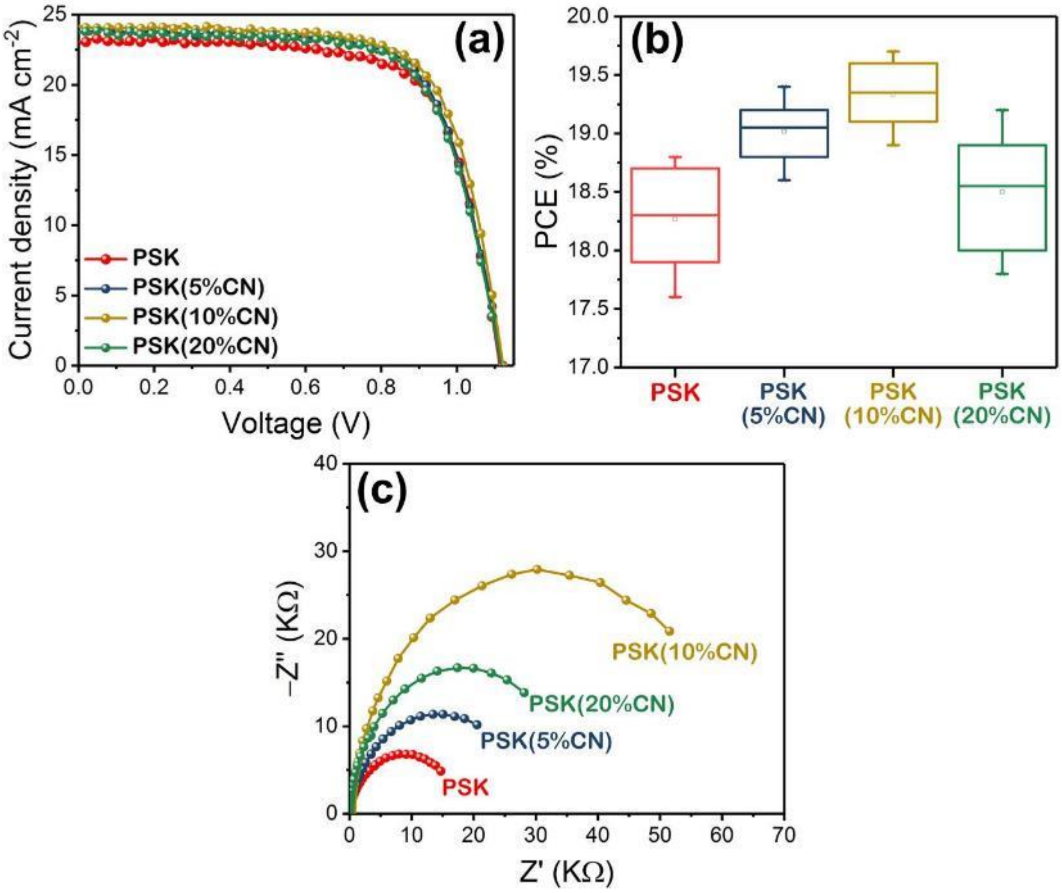

Figure S24. a) *J*–*V* curves under simulated AM 1.5 illumination and b) PCE distributions (25 devices are fabricated for each sample) and c) EIS plots recorded under dark condition for PSCs based on different concentrations of CN surface modification.

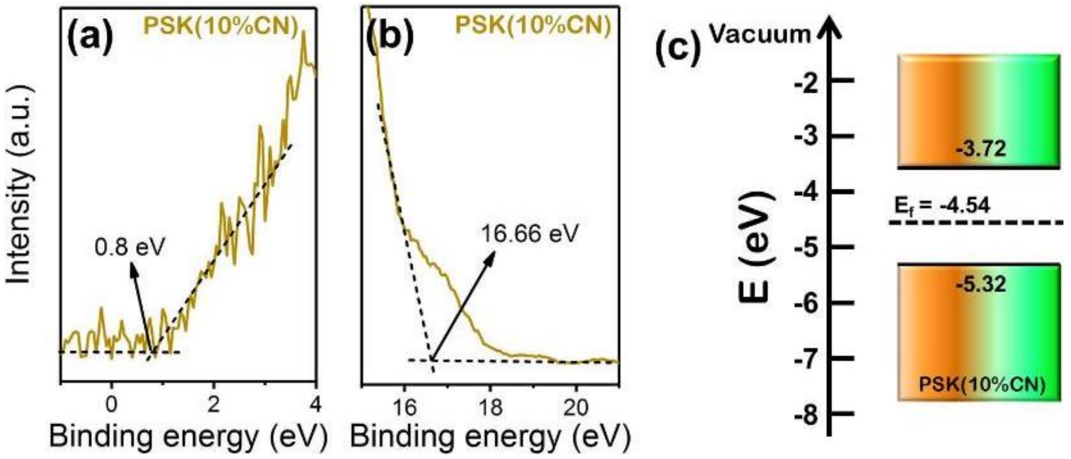

Figure S25. UPS spectra of a,b) PSK(10%CN). c) The position distribution of conduction band (CB), valence band (VB) and Fermi level (E_f) in PSK(10%CN).

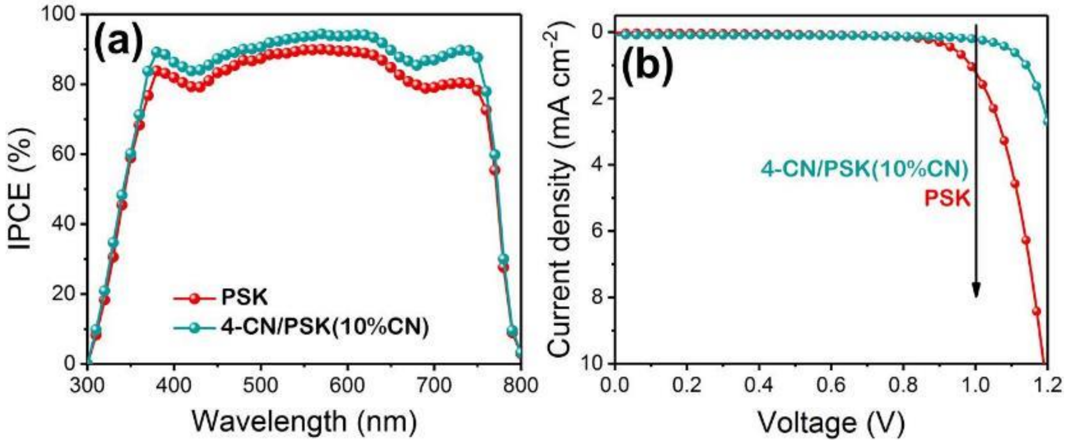

Figure 26. a) IPCE spectra and b) dark *J*-*V* curves for PSK and 4-CN/PSK(10%CN) based PSCs.

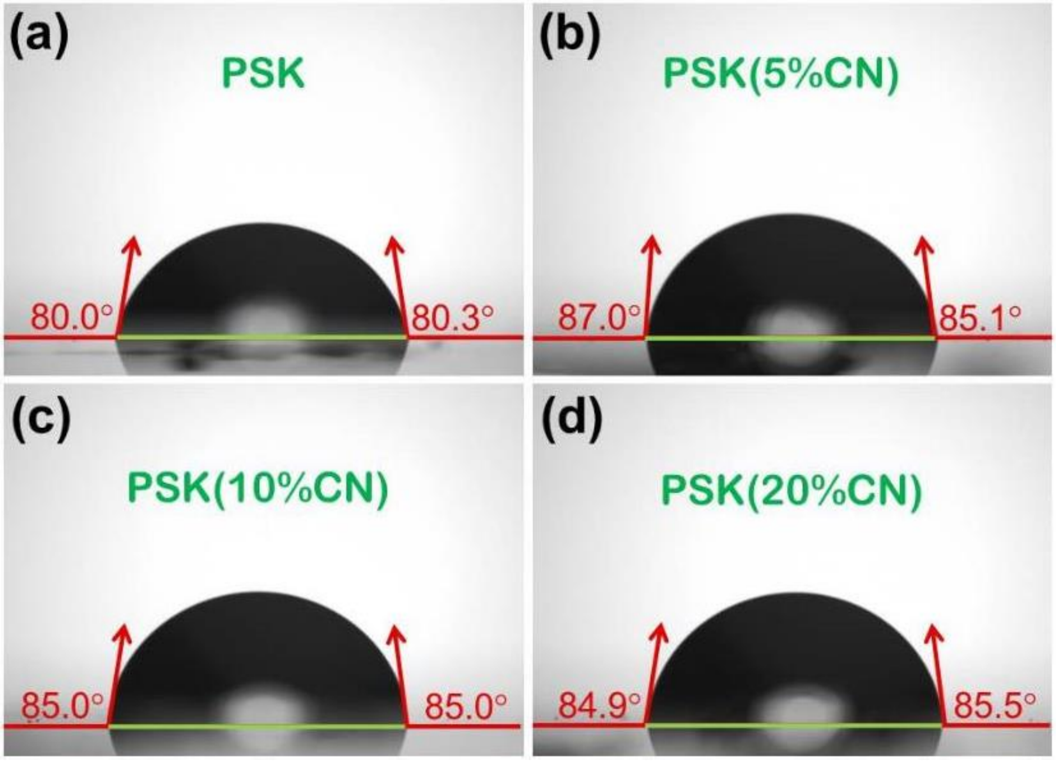

Figure S27. a–d) The contact angles of water droplets on perovskite films synthesized through antisolvent containing different concentrations of CN.

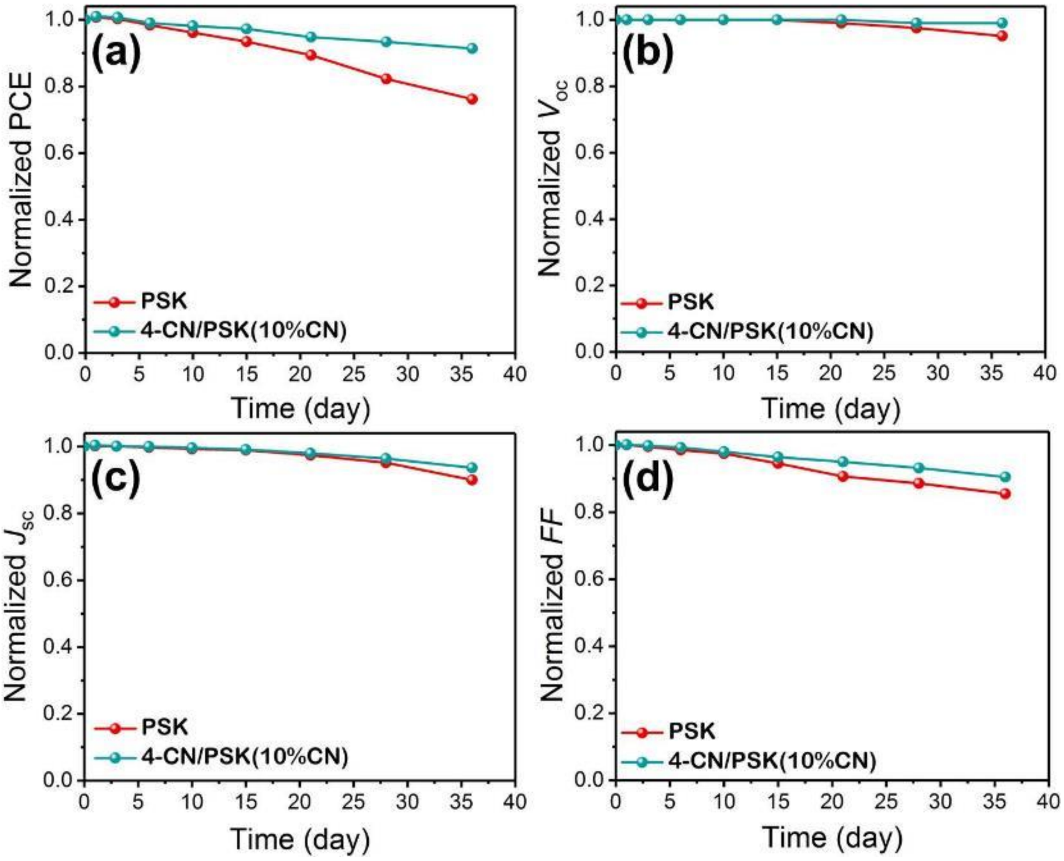

Figure S28. a–d) The long-term stability test of PSCs stored in an air atmosphere with humidity of 60% ± 5%.

Table S1. Transient absorption kinetics parameters for FTO/SnO_2/PSK and FTO/SnO_2/CN/PSK at 759 nm.

Samples	A_1	τ_1 [ps]	A_2	τ_2 [ps]	τ_{ave} [ps]
FTO/SnO_2/PSK	0.400	611	0.600	3570	3266
FTO/SnO_2/CN/PSK	0.423	258	0.577	2720	2560

The results are fitted with the following double exponential equation:

$$\mathrm{y} = y_0 + A_1 \exp\left(-\frac{t}{\tau_1}\right) + A_2 \exp(-\frac{t}{\tau_2})$$

The average decay time is calculated through the following format:

$$\tau_{ave} = \frac{A_1 * \tau_1^2 + A_2 * \tau_2^2}{A_1 * \tau_1 + A_2 * \tau_2}$$

Table S2. Detailed photovoltaic parameters for PSCs based on different times of CN bottom modification (25 devices are fabricated for each sample).

Samples	V_{oc} [V]	J_{sc} [mA cm^{-2}]	*FF* [%]	PCE [%]
PSK	1.11 ± 0.01	23.07 ± 0.37	71.1 ± 1.6	18.2 ± 0.6
2-CN/PSK	1.11 ± 0.01	23.88 ± 0.26	71.3 ± 1.4	18.9 ± 0.5
4-CN/PSK	1.12 ± 0.01	24.16 ± 0.18	71.7 ± 1.3	19.4 ± 0.4
6-CN/PSK	1.12 ± 0.01	24.12 ± 0.16	70.0 ± 2.1	18.9 ± 0.6

Table S3. Transient absorption kinetics parameters for PSK and PSK(CN) at 759 nm.

Samples	A_1	τ_1 [ps]	A_2	τ_2 [ps]	τ_{ave} [ps]
FTO/SnO_2/PSK	0.515	681	0.485	5003	3266
FTO/SnO_2/PSK(CN)	0.563	705	0.437	6010	5313

The results are fitted with the following double exponential equation:

$$y = y_0 + A_1 \exp\left(-\frac{t}{\tau_1}\right) + A_2 \exp(-\frac{t}{\tau_2})$$

The average decay time is calculated through the following format:

$$\tau_{ave} = \frac{A_1 * \tau_1^2 + A_2 * \tau_2^2}{A_1 * \tau_1 + A_2 * \tau_2}$$

Table S4. Detailed photovoltaic parameters for PSCs based on different concentrations of CN surface modification (25 devices are fabricated for each sample).

Samples	V_{oc} [V]	J_{sc} [mA cm^{-2}]	*FF* [%]	PCE [%]
PSK	1.11 ± 0.01	23.07 ± 0.37	71.1 ± 1.6	18.2 ± 0.6
PSK(5%CN)	1.12 ± 0.01	23.80 ± 0.23	71.3 ± 1.2	19.0 ± 0.4
PSK(10%CN)	1.12 ± 0.01	24.08 ± 0.24	71.6 ± 1.1	19.3 ± 0.4
PSK(20%CN)	1.12 ±0.01	23.72 ± 0.34	70.0 ± 1.4	18.5 ± 0.7

References:

[S1] Q. Akkerman, M. Gandini, F. Di Stasio, P. Rastogi, F. Palazon, G. Bertoni, J. Ball, M. Prato, A. Petrozza, L. Manna, *Nat. Energy* **2017**, *2*, 16194.
